# Metabolic and genetic analysis links *TRITERPENE SYNTHASE 12* to oleanolic acid biosynthesis in grape berry wax

**DOI:** 10.1093/jxb/eraf119

**Published:** 2025-03-18

**Authors:** Jessica A Vervalle, Melané A Vivier, Jos D Cox, Boje Müller, Christian Schulze Gronover, Ken R Tobutt, Phyllis Burger, Rouvay Roodt-Wilding, Justin G Lashbrooke

**Affiliations:** Department of Genetics, Stellenbosch University, Stellenbosch 7600, South Africa; ARC Infruitec-Nietvoorbij, Stellenbosch 7599, South Africa; South African Grape and Wine Research Institute, Stellenbosch University, Stellenbosch 7600, South Africa; Fraunhofer Institute for Molecular Biology and Applied Ecology IME, D-48143 Münster, Germany; Fraunhofer Institute for Molecular Biology and Applied Ecology IME, D-48143 Münster, Germany; Fraunhofer Institute for Molecular Biology and Applied Ecology IME, D-48143 Münster, Germany; ARC Infruitec-Nietvoorbij, Stellenbosch 7599, South Africa; ARC Infruitec-Nietvoorbij, Stellenbosch 7599, South Africa; Department of Genetics, Stellenbosch University, Stellenbosch 7600, South Africa; Department of Genetics, Stellenbosch University, Stellenbosch 7600, South Africa; The James Hutton Institute, UK

**Keywords:** Amyrin synthase, berry development, cuticular wax, fruit surface wax, grapevine, oleanolic acid, quantitative trait loci, triterpene synthase, triterpenoid, *VvTTPS12*

## Abstract

Fruit surface cuticular waxes of grape berries are important in stress response and fruit quality. Despite extensive studies on the biosynthesis, regulation, and composition of fruit surface waxes, knowledge of the compositional variation and genetic mechanisms underlying grape berry cuticular wax formation remains limited. This study aimed to characterize grape berry cuticular wax composition and identify contributing genes. The wax composition of two grape cultivars (‘Deckrot’ and G1-7720) and their progeny shifted from aldehyde to fatty acid accumulation during ripening, while the composition was shown to influence *Botrytis cinerea* susceptibility. Alcohols and aldehydes contributed to the glaucous wax appearance, while the bioactive triterpene, oleanolic acid, was found to be the most abundant wax monomer. Metabolic quantitative trait locus analysis identified several genomic regions associated with wax monomer formation, including a cluster on chromosome 9 linked to triterpene content, which included eight putative triterpene synthases. Molecular phylogenetic analysis suggested that these genes code for amyrin synthases. Co-expression analysis, and subsequent heterologous expression in yeast, confirmed the involvement of *VvTTPS12* in oleanolic acid formation. This study explores the role of grape berry wax composition and enhances understanding of genetic contributors to wax formation.

## Teaser

Metabolic quantitative trait locus analysis reveals the genetic architecture underlying grape berry wax formation and identifies *VvTTPS12* as a β-amyrin synthase contributing to the formation of the triterpene oleanolic acid.

## Introduction

The plant cuticle, which protects against external stresses, consists of a polymer cutin matrix and soluble lipids known as cuticular waxes (Riederer and Schreiber, 2001; Reina-Pinto and Yephremov, 2009; González Moreno *et al.*, 2022). Most studies have focused on leaf waxes, but there has been recent interest in fruit surface waxes because of their importance in fruit quality, post-harvest shelf life, and disease infection (Jetter *et al.*, 2006; Martin and Rose, 2014). Detailed knowledge on the synthesis, regulation, and composition is essential both to understand the function of these surface waxes and to allow for the identification of targets and strategies towards plant improvement (Jolliffe *et al*., 2023).

Until now the vast majority of cuticle research has been performed in model systems such as tomato and Arabidopsis, despite the fact that the composition of surface waxes varies greatly between different species, and also between different organs and their developmental stages. Cuticular waxes consist of a mixture of long chain aliphatics (fatty acids and their derivatives) as well as secondary metabolites, such as triterpenoids. The long chain aliphatics form the ordered crystalline structure of the cuticle, whereas the secondary metabolites form clusters outside the crystalline structure and contribute to the amorphous character of the cuticle (Casado and Heredia, 1999). Knowledge of the composition of the fruit surface waxes is important, as the chemistry and structure determine the barrier function of the cuticle, such as cuticular transpiration and resistance against pathogens. The composition of fruit surface waxes has predominantly been studied in apple and tomato, in which they consist mainly of the long chain aliphatics. In other fruits, such as the olive (Bianchi *et al.*, 1992), persimmon (Tsubaki *et al.*, 2013), blueberry (Chu *et al.*, 2017), and grape, triterpenoids predominate, with significantly fewer long chain aliphatics. The substantial variation in surface waxes underscores the need to investigate the composition and synthesis of waxes in different fruit species.

Studies carried out predominantly in Arabidopsis and tomato have provided a general description of cuticular wax biosynthesis. Aliphatic wax synthesis involves the production of long chain fatty acids and their derivatives including alkanes, aldehydes, alcohols, and esters. In the epidermal cells of fruit skins, C16 and C18 fatty acids are produced in the plastids, using acetyl-CoA as a building block. Thereafter, these fatty acids are elongated to C20–C34 fatty acids, also called the very long chain fatty acids (VLCFAs), in the endoplasmic reticulum. This elongation is carried out by the multienzymatic fatty acid elongase (FAE) complex (Bernard and Joubès, 2013). Lastly, the resulting VLCFAs are converted to aldehydes, alkanes, secondary alcohols, or ketones in the decarbonylation pathway, or to primary alcohols and wax esters in the acyl reduction pathway (Trivedi *et al*., 2019). In the decarbonylation pathway, VLCFAs are converted to aldehydes and alkanes by CER1, CER3, and CytB5 complexes (Rowland *et al*., 2007; Bernard *et al*., 2012), and to secondary alcohols and ketones by midchain alkane hydroxylase 1 (MAH1). Alternatively, in the acyl reduction pathway, the fatty acyl-CoA reductase (CER4/FAR3) and wax synthase/diacylglycerol acyltransferase (WSD1) convert VLCFAs to alcohols and wax esters, respectively (Rowland *et al*., 2006). The synthesized wax aliphatics are then transported across the plasma membra to the extracellular surface by ATP-binding cassette (ABC) transporters (CER5 and ABCG; Bird *et al*., 2007) and lipid transfer proteins (LTPs; Kim *et al*., 2012).

In cuticular waxes, oleanolic acid and ursolic acid are common triterpenoids. Triterpenoids are biologically active compounds displaying antifungal, antibacterial, and anti-inflammatory effects (Sparg *et al.*, 2004). Plants synthesize a huge array of structurally diverse triterpenoids via the mevalonate pathway. The synthesis of these diverse triterpenoids starts with a common precursor, 2,3-oxidosqualene. This substrate is cyclized by oxidosqualene cyclases (OSCs), which can be divided into sterol synthases or triterpene synthases. Triterpene synthases are responsible for producing triterpenoid scaffolds, such as α-amyrin and β-amyrin. Subsequently, these triterpenoid scaffolds are modified by various cytochrome P450 monooxygenases (P450s) to form triterpenoids. Several OSCs and P450s involved in triterpenoid biosynthesis exist in higher plants, allowing the synthesis of a vast array of triterpenoids. In grapevine, the triterpenoid oleanolic acid is a predominant compound of the berry cuticular wax (Radler, 1965; Commenil *et al*., 1997; Rid *et al*., 2018). Oleanolic acid is synthesized from the triterpenoid scaffold β-amyrin. Several potential triterpene synthases have been identified in grape (Pensec *et al*., 2016), but have not yet been linked to the synthesis of β-amyrin. The CYP716A12 enzyme has been identified in *Medicago truncatula* as a β-amyrin 28-oxidase able to modify β-amyrin to oleanolic acid (Fukushima *et al*., 2011). Two homologues, CYP716A15 and CYP716A17, have been identified and functionally characterized in grapevine.

The deposition of the cuticular waxes on the extracellular surface presents as a whitish surface coating often described as glaucous, in contrast to the glossy phenotype. It is assumed that the glaucous phenotype is associated with a higher wax load. The berry wax composition of different grape cultivars has been investigated (Yamamura and Naito, 1983; Yang *et al*., 2021; Zhang *et al*., 2021) and varying amounts of wax deposition between cultivars have been found. However, the chemotype of the glossy and glaucous wax phenotypes has not yet been investigated. Chemotypes can seem similar in morphology but differ in their composition of secondary metabolites, such as the wax compounds. A mapping population that arose from the cross between ‘Deckrot’ and G1-7720 presents a valuable tool to study the visual wax phenotypes. ‘Deckrot’ is a teinturier grape variety with a very glaucous phenotype, whereas G1-7720 is a table grape accession with a glossy phenotype. Understanding the diversity of wax composition is crucial for uncovering the role and function of these waxes.

This study will characterize the metabolic and genetic aspects of the cuticular wax of grapevine berries. First, the role of the cuticular waxes in *Botrytis cinerea* infection in grape berries will be investigated. Second, the composition of the grape berry waxes will be determined in order to understand how the waxes are formed and contribute to the glaucous and glossy phenotypes. Lastly, the molecular basis of the grape berry cuticular waxes will be explored to identify possible candidate genes that contribute to wax synthesis in grape berries. This novel knowledge on the function, composition, and molecular basis will help to better understand and exploit the natural plant defence mechanism of grapevine.

## Materials and methods

### Plant material and sampling

**Fig. 1.**
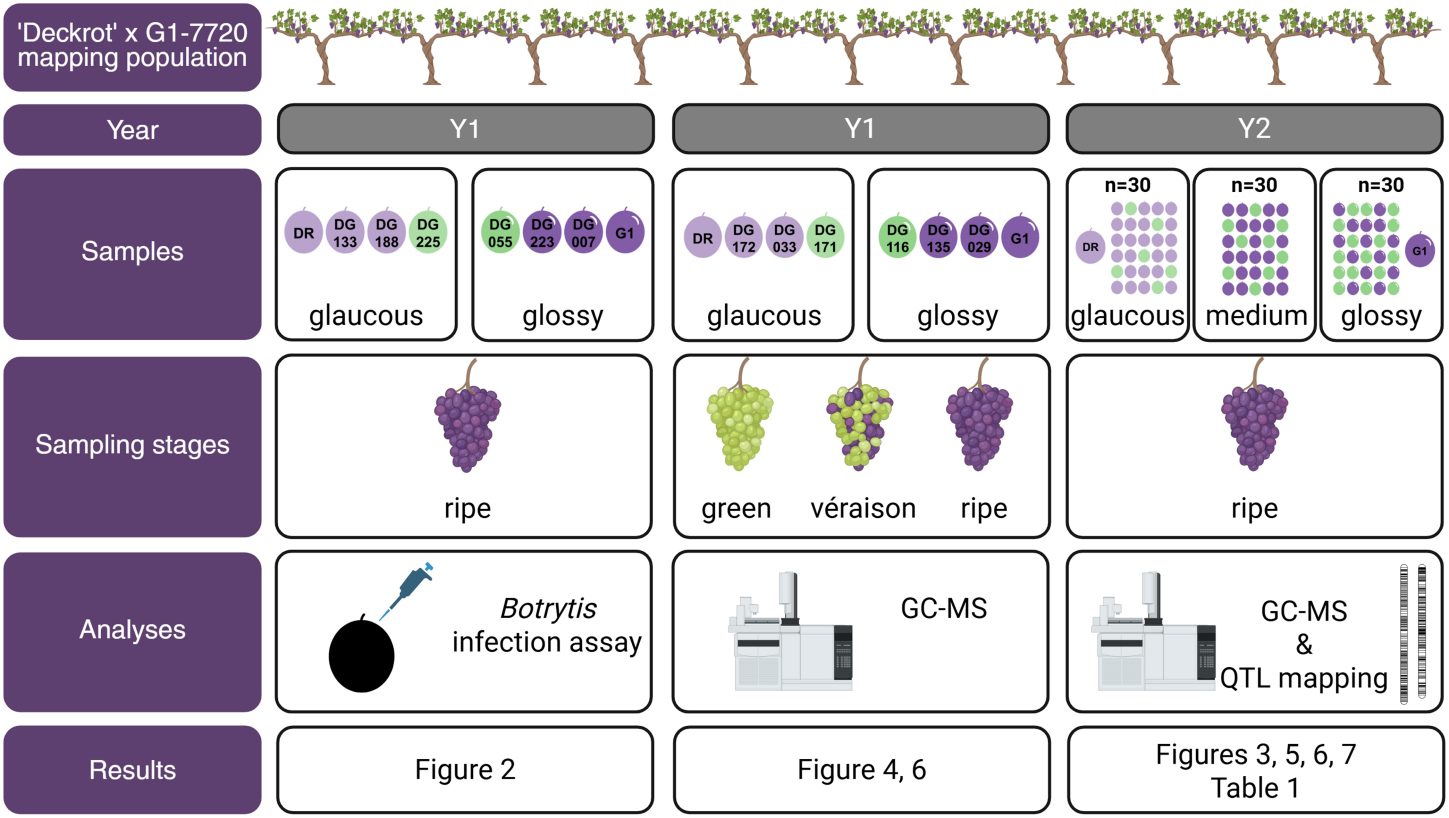
Sampling strategy utilized in this study. Overview of the strategy used to sample the grape population ‘Deckrot’×G1-7720 indicating the sampling year (Y1 or Y2), number and description of individuals, and the phenological developmental stages of sampling. Furthermore, the analyses and results for which these samples were used are provided. Berry colour segregated in the progeny: white berries are indicated in green (e.g. DG225) and black berries are indicated in purple (e.g. DG133). Image created in BioRender.com/x3015jc. DR, ‘Deckrot’; G1, G1-7720; QTL, quantitative trait locus.

Grape berries were sampled from grapevine (*Vitis vinifera*) cultivars ‘Deckrot’ and G1-7720 and the progeny from the cross ‘Deckrot’×G1-7720. ‘Deckrot’ results from the cross ‘Pinot Gris’×‘Teinturier’, and the berries show a very glaucous phenotype. G1-7720 comes from the cross ‘Black Rose’×‘Muscat Seedless’ made by the Agricultural Research Council (ARC) table grape breeding programme, and the berries are glossy. The progeny were planted in 2011 in a single block at ARC-Nietvoorbij, South Africa (33°54'47.6''S, 18°51'54.9''E). The offspring were phenotyped and classified as glossy, medium, or glaucous on the basis of visual wax appearance scored according to OIV descriptor 227 (Organisation Internationale de la Vigne et du Vin, 2009). For this study, berries were sampled at different phenological developmental stages over two seasons: 2014/2015 (year 1, Y1) and 2015/2016 (year 2, Y2). An overview of the sampling strategy is presented in Fig. 1, with details in the sections below. Weather data for these two seasons were obtained from the Helderfontein weather station (33°55'23.9''S, 18°52'23.9''E), located 1.3 km from the vineyard.

### *Botrytis cinerea* infection assay

Berries were sampled from the glossy and glaucous parents and select offspring (Fig. 1; Supplementary Table S1). Bunches were harvested during Y1 from the vines in the morning at stage E-L38 on the Eichorn and Lorenz growth scale (Coombe, 1995). Uniform and healthy looking grape berries were selected and individually separated from an intact bunch for each sample. Six of these berries were submerged for 10 s in chloroform to remove the waxy layer of the cuticle (Marois *et al.*, 1986) and subsequently inoculated with *B. cinerea*; this treatment was designated wax–. The waxy layer of the remaining berries was left intact, and six berries were inoculated with *B. cinerea*, treatment wax+, and six berries were not inoculated as the negative control.

Berries were placed on sterile 6-well tissue culture plates and inoculated with *B*. *cinerea* spores as described by Weiller *et al*. (2021). Spores were diluted to a concentration of 200 spores µl^–1^ using 25% sterile red grape juice (Liquifruit, Pioneer Foods, Paarl, South Africa). For the infection treatment, each berry was inoculated with a 5 µl droplet containing 1000 spores without creating wounds. Negative controls were inoculated with 5 µl of 25% sterile red grape juice. Berries were observed for infection symptoms on 1, 2, 4, and 8 days post-infection (dpi). Fungal mycelial growth was scored as: 0 (no mycelia observed); 1 (mycelia observed around the infection spot); 2 (>30% of the top of the berry covered with mycelia); 3 (>50% of the top of the berry covered with mycelia); 4 (top of the berry completely covered with mycelia); or 5 (top of the berry completely covered with dense mycelia). Other infection indicators such as leaking of fluid, discoloration due to necrosis, and skin lacerations were scored as 0 (absent) or 1 (present).

### Grape berry cuticle isolation

Cuticle discs were isolated from the berries using a modified version of the method described by Fernandez-Moreno *et al*. (2016). In Y1, berries were sampled from glossy and glaucous parents and select offspring (*n*=6; Fig. 1; Supplementary Table S1) at stage E-L33, E-L35, and E-L38. In Y2, berries were sampled from both parents and 90 offspring at stage E-L38 (Fig. 1; Supplementary Table S1). Each biological replicate consisted of 10 skin discs of 5 mm radius (total area: 785.40 mm^2^) which were punched from the skin of 10 individual fruit from a single cluster. Skin discs were placed in enzymatic buffer (1% pectinase, 0.5% cellulose, 1 mM sodium azide, 0.1 M sodium acetate, pH 3.8) and incubated at 37 °C for 2 d with shaking. Cuticles were washed with distilled water and placed into fresh enzymatic buffer for an additional day (or until the pericarp was completely digested). Cuticles were washed with a 10 mM borate buffer (pH 9.2) and rinsed with distilled water before drying at 37 °C and storing at room temperature.

### Cuticular wax isolation and GC-MS analysis

Cuticular waxes were extracted from the isolated cuticles by submerging 10 discs per biological replicate twice into 2 ml of chloroform for 1 min at room temperature (*n*=3). A 10 μg aliquot of the internal standard cholestanol (Sigma) was added to each wax extraction. Samples were completely evaporated under nitrogen, followed by resuspension in 100 µl of chloroform. Samples were then derivatized using *N*,*O*-bis(trimethylsilyl)trifluoroacetamide (BSTFA; Sigma) in pyridine at 70 °C for 40 min before injection. Profiling of the extracted wax was performed using GC-MS on an Agilent 6890N gas chromatograph coupled to an Agilent 5975C mass spectrometer (Agilent Technologies, CA, USA) using a Zebron ZB-SemiVolatiles (30 m×0.25 mm×0.25 µm, Phenomenex). Sample volumes of 1 µl were injected with a split ratio of 10:1 at 100 °C and held for 2 min; temperature was then increased to 180 °C at 15 °C min^–1^, raised to 250 °C at 5 °C min^–1^, held at 250 °C for 3 min, raised to 320 °C at 20 °C min^–1^, and held at 320 °C for 20 min. The flow rate of the helium carrier gas was 9.9 ml min^–1^. Reproducibility of compound retention was assessed by comparing the peak times of a standard mix of alkanes (C7–C40 alkanes standard, Sigma) at the start of each sequence of samples.

Wax compounds were identified by comparing mass spectra and retention indexes with data from the literature as detailed by Fernandez-Moreno *et al*. (2016). Compounds were quantified using Xcalibur™ software (ThermoFisher Scientific) and were normalized to the peak area of the internal standard (10 µg) and the total extraction surface area (785.40 mm^2^) to estimate the absolute abundance (ng mm^–2^) of each compound. The wax load (total wax) was estimated by the sum of the absolute abundances of all compounds. Subsequently, the relative abundance of each compound was determined as a percentage of the total wax. The total abundance of each compound class (fatty acids, alkanes, alcohols, aldehydes, triterpenoids, and unknown) was calculated, as were the ratios between classes. Lastly, the compounds were grouped according to chain length to determine the abundance of each chain length (C16, C18, C20, C22, C24, C25, C26, C27, C28, C29, C30, C31, and C32).

### Statistical analysis

The *B*. *cinerea* infection assay was conducted on glossy and glaucous berries in Y1. Glossy berries were obtained from two plants of the parent G1-7720 and three glossy progeny (Fig. 1; Supplementary Table S1). Glaucous berries were obtained from two plants of the parent ‘Deckrot’ and three glaucous progeny. Eighteen berries were sampled from each individual, and divided over the wax– treatment (*n*=6), the wax+ treatment (*n*=6), and the negative control (*n*=6). The experiment was performed twice. Comparisons of infection indicators between glaucous and glossy berries were tested with the Mann–Whitney U-test (mycelial growth) or Fisher’s exact test (other infection indicators). Comparisons between wax– and wax+ treatments were tested with the Wilcoxon signed rank test. Statistical analyses were performed with XLSTAT (v2015.4.01.20575). The wax composition was compared during berry development in Y1 and between glossy and glaucous phenotypes in Y2. For berry development, in Y1, berries were sampled from the parents (two plants each of ‘Deckrot’ and G1-7720) and six progeny, at green (E-L33), véraison (E-L35), and ripe (E-L38) stages (Fig. 1; Supplementary Table S1). Three replicates of 10 skin discs each were isolated and analysed independently. For waxy phenotypes, in Y2, berries were sampled from the glossy (parent G1-7720 and 30 progeny), medium (30 progeny), and glaucous (parent ‘Deckrot’ and 30 progeny) plants. Thirty skin discs were enzymatically isolated and divided over three biological replicates of 10 discs each for GC-MS analysis. Principal component analysis (PCA) was performed with SIMCA (v17.0.2) to identify the main contributors to differentiation. Subsequently, data were visualized using the ggplot2 package (Wickham, 2011).

### Quantitative trait locus mapping

Genetic linkage maps were previously created for the parents using simple sequence repeat (SSR) and single nucleotide polymorphism (SNP) markers (Vervalle *et al.*, 2022). The genetic map for ‘Deckrot’ contained 1910 markers with an interlocus distance of 0.79 cM, whereas the G1-7720 map contained 2252 markers with an interlocus distance of 0.82 cM. These parental maps were used to perform quantitative trait locus (QTL) analyses in MapQTL® 6 (Van Ooijen, 2009). The absolute abundances of each compound in 90 progeny (Supplementary Table S1) were used as quantitative trait data. These 90 progeny consisted of 30 glossy and 30 glaucous phenotypes, and an additional 30 medium wax phenotypes (Fig. 1). The genome-wide significance logarithm of odds (LOD) threshold for each trait at *P*≤0.05 was determined using a permutation test with 1000 permutations. Preliminary QTL regions were identified using interval mapping (IM). QTL regions were then further specified using multiple QTL mapping (MQM) analysis. In this step, the marker with the highest LOD value during IM was selected as a cofactor for MQM, helping to reduce the residual variance of the QTL region.

### Genomic analysis of significant quantitative trait locus regions

The physical positions of the significant QTL regions were obtained from the grapevine PN40024v.2 reference genome (Jaillon *et al.*, 2007) by using the position of the two flanking markers. Lists of annotated genes for these regions were retrieved from URGI (https://urgi.versailles.inra.fr/Species/Vitis/Annotations) based on the VCost.v3 structural annotation of the reference genome (Canaguier *et al.*, 2017). Gene function was predicted using BLASTp analysis by querying protein sequences against the UniProtKB/SwissProt database and selecting the top hit for each sequence. Based on these annotated gene functions, possible candidate genes were selected using evidence from the literature. The function of the β-amyrin synthase candidate genes was further investigated through molecular phylogenetic analysis. Homologous genes in the grapevine genome were identified using a BLAST search in the VCost.v3 genome annotation. Gene sequences with >2000 nucleotides were included. Amino acid sequences were then obtained from the NCBI and aligned to construct a Neighbor–Joining tree based on 1000 bootstrap replicates with Clustal X v2.0 (Larkin *et al.*, 2007). Phylogenetic trees were visualized and edited in Treeviewer v2.2.0 (https://treeviewer.org/).

### Gene expression analysis

The VitViz platform, which uses expression data from public databases, was used to investigate further the gene expression profile of candidate genes (Orduña *et al.*, 2023). Expression levels of genes during berry development were studied in ‘Pinot Noir’ and ‘Cabernet Sauvignon’ using the Expression Atlases app. A gene co-expression network of candidate amyrin synthases was constructed using the tissue-independent database in the AggCGNs app.

### Heterologous expression of triterpene synthases in *Saccharomyces cerevisiae*

The sequence of the *VvTTPS12* gene was codon harmonized for *S. cerevisiae* using CODON HARMONIZER (http://biocatalysis.uni-graz.at/sites/codonharmonizer.html). The resulting sequences were synthesized by GeneArt (Thermo Fisher Scientific, Waltham, MA, USA) with *Kpn*I/*Xho*I restriction sites inserted into Gateway vector pENTR3c and introduced into pAG304_P_Gal1_-ccdB (Alberti *et al.*, 2007) via LR recombination (Thermo Fisher Scientific). The resulting constructs were linearized with *Bsg*I cutting in the tryptophan (*trp1*) auxotrophic marker. The *S. cerevisiae* strain CEN.PK2-1C rox1::P_GAL1_-tHMGR P_GAL10_-ERG13 P_ERG7Δ_::P_CTR3_ previously described and optimized for triterpenoid synthesis by Bröker *et al.* (2018) was transformed with the prepared constructs using the lithium acetate method (Gietz and Schiestl, 2007). Triterpenoid production of yeast strains was assayed as described by Pütter *et al*. (2019). Strains were inoculated into 5 ml of yeast extract peptone dextrose (YPD) medium and incubated overnight at 30 °C. From there, a 50 ml YPD main culture containing 150 µM CuSO_4_ was inoculated to an OD_600_ of 0.2 in a 250 ml Erlenmeyer flask and incubated at 30 °C and 8 *g*. At an OD_600_ of 0.4, the culture medium was switched to extract peptone galactose medium (containing 150 µM CuSO_4_), inducing expression of genes controlled by P_Gal1_ and P_Gal10_, and further incubated for 96 h. Yeast cells were harvested by centrifugation (10 min at 1000 *g*), and pellets were freeze-dried.

### Triterpenoid extraction and GC-MS analysis

The freeze-dried cell pellets were ground (5 min at 30 Hz) in 2 ml reaction tubes containing 500 µl of glass beads (0.5–2.7 mm; Carl Roth, Karlsruhe, Germany), 1 ml of ethyl acetate, and 100 µg of cholestanol as internal standard, using the Mixer Mill MM400 (Retsch, Haan, Germany). Samples were centrifuged for 2 min at 20 000 *g*, and the upper phase was transferred to a glass vial. The extraction was performed three times, and the pooled extracts were evaporated. Samples were re-solubilized in 1 ml of acetone and filtered (0.45 µm) prior to GC-MS analysis. GC-MS was performed as described by Bröker *et al*. (2018) using a GC-MS-QP 2010 Ultra (Shimadzu, Duisburg, Germany) and a 30 m Rtx-5MS column. Peak areas of total ion counts were integrated and normalized against the internal cholestanol standard.

## Results

### Cuticular waxes influence *Botrytis cinerea* susceptibility in a genotype-dependent manner

**Fig. 2.**
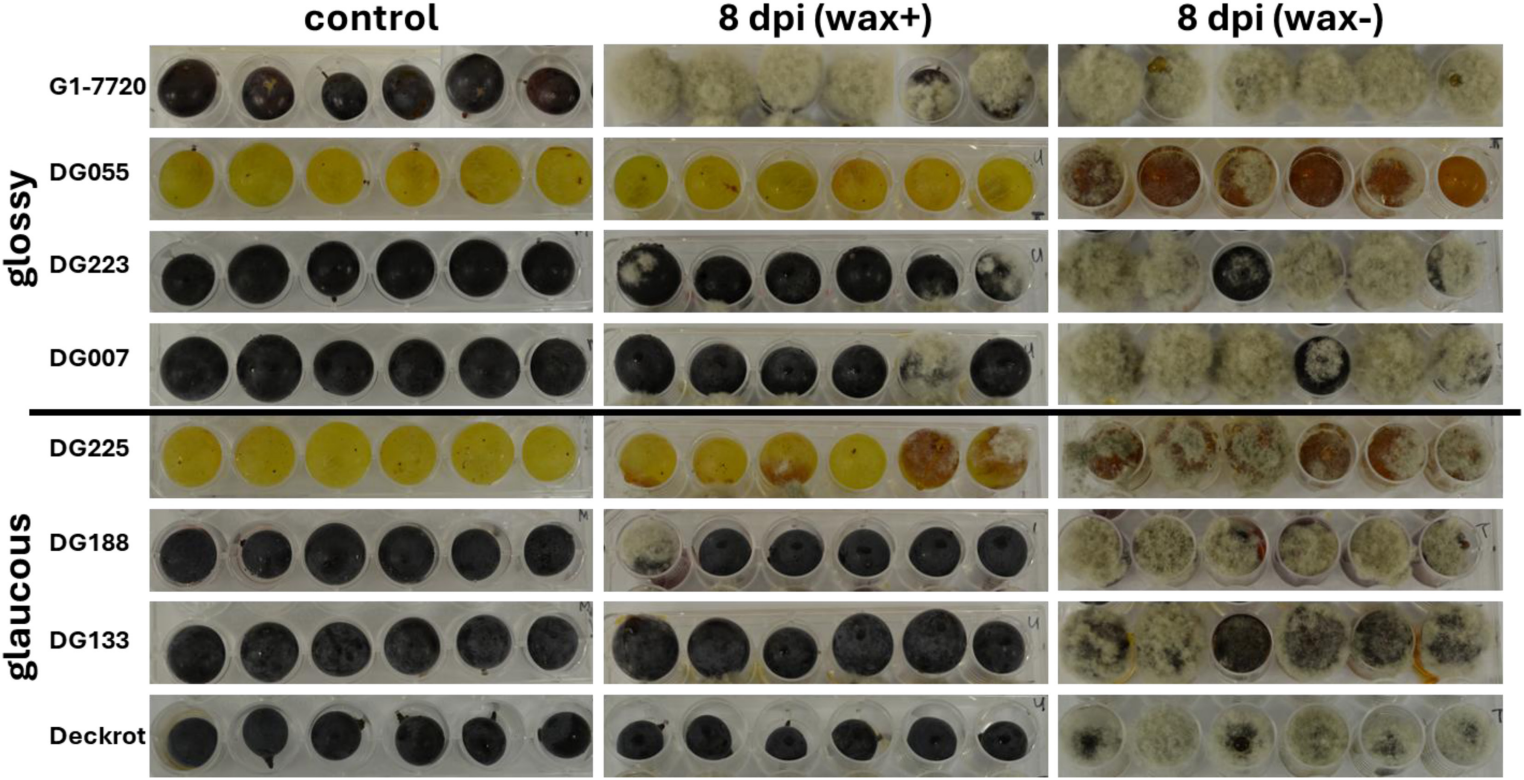
*B. cinerea* infections of grape berries. Grape berries of the parents (‘Deckrot’ and G1-7720) and three glossy offspring individuals (DG055, DG223, and DG007) and three glaucous offspring individuals (DG225, DG188, and DG133) infected with *B. cinerea* with wax intact (wax+) or wax removed (wax–) at 8 days post-infection (dpi). Infection of wax+ individuals at 0, 2, 3, and 4 dpi is shown in Supplementary Fig. S2. The control treatment shows uninoculated berries with wax intact.

*Botrytis cinerea* infection assays were performed on ripe berries of the parents, ‘Deckrot’ and G1-7720, and six selected offspring; fungal infection in berries with intact wax (wax+) was compared with berries from which the cuticular waxes were removed (wax–). Mycelial growth was significantly greater in wax– berries than in wax+ berries from 1 dpi (*P*=0.0013; Supplementary Fig. S1). At 8 dpi, all wax– berries exhibited severe *B*. *cinerea* infections (Fig. 2) whereas control berries, with waxes intact and uninoculated, showed no symptoms. Removal of the wax layer increased the susceptibility of berries to *B*. *cinerea* infection, as infection occurred sooner and was more severe in wax– berries.

In the case of berries with intact wax, no infection was observed in the glaucous parent ‘Deckrot’ for the entire time course of the experiment, whereas, in the glossy parent G1-7720, infections were observed from 2 dpi, and by 8 dpi all the berries were infected (Supplementary Fig. S2), indicating a difference in susceptibility between the two. In the offspring, infections progressed more slowly than in the G1-7720 parent, with most infections only visible at 8 dpi. Susceptibility amongst wax phenotypes varied; of the glaucous progeny, two showed no to minimal infection (DG133 and DG188) and one showed severe infection (DG225), whereas of the glossy progeny one showed no infection (DG055), one minimal infection (DG223), and one severe infection (DG007).

### Grape berry cuticular wax consists predominantly of the triterpenoid oleanolic acid

**Fig. 3.**
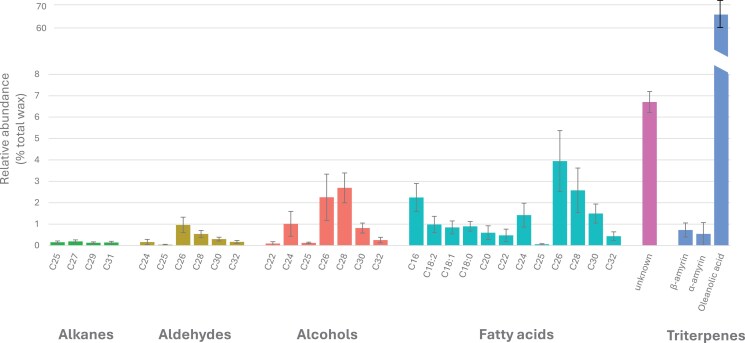
Grape berry cuticular wax composition. Average composition of the grape berry cuticular wax based on the chemotyping of the two parents (‘Deckrot’ and G1-7720) and 90 progeny (glossy, medium, and glaucous). Compounds were classed as: alkanes, aldehydes, alcohols, fatty acids, unknowns, and triterpenes, and arranged according to the chain length of the compound. Abundance is displayed as a percentage of the total amount of wax. Error bars indicate the SD of the compound across all samples.

The constituent compounds of the fruit cuticular wax layer of ripe berries were determined for the parents and 90 offspring (glossy, medium, and glaucous) via GC-MS. A total of 47 peaks were identified in the wax extracted, and included alcohols (C22, C24, C25, C26, C28, C30, C32), aldehydes (C24, C25, C26, C28, C30, C32), alkanes (C25, C27, C29, C31), fatty acids (C16, C18:0, C18:1, C18:2, C20, C22, C24, C25, C26, C28, C30, C32), triterpenoids (α- and β-amyrin, oleanolic acid, and derivatives of oleanolic acid), as well as nine as yet unidentified (unknown) compounds (Supplementary Table S2). The cuticular wax consisted mostly of triterpenoids, which constituted on average >70% of the total (Fig. 3). Oleanolic acid was the most abundant compound of the cuticular wax, contributing on average to 65% of the total wax. Fatty acids and alcohols were the second most abundant groups of compounds, making up on average 16% and 7% of the total wax composition, with C26 fatty acid and C28 alcohol being the most prominent. The least abundant compounds were alkanes with <1% of the wax composition.

### Wax composition shifts from aldehydes to fatty acids during grape berry development

**Fig. 4.**
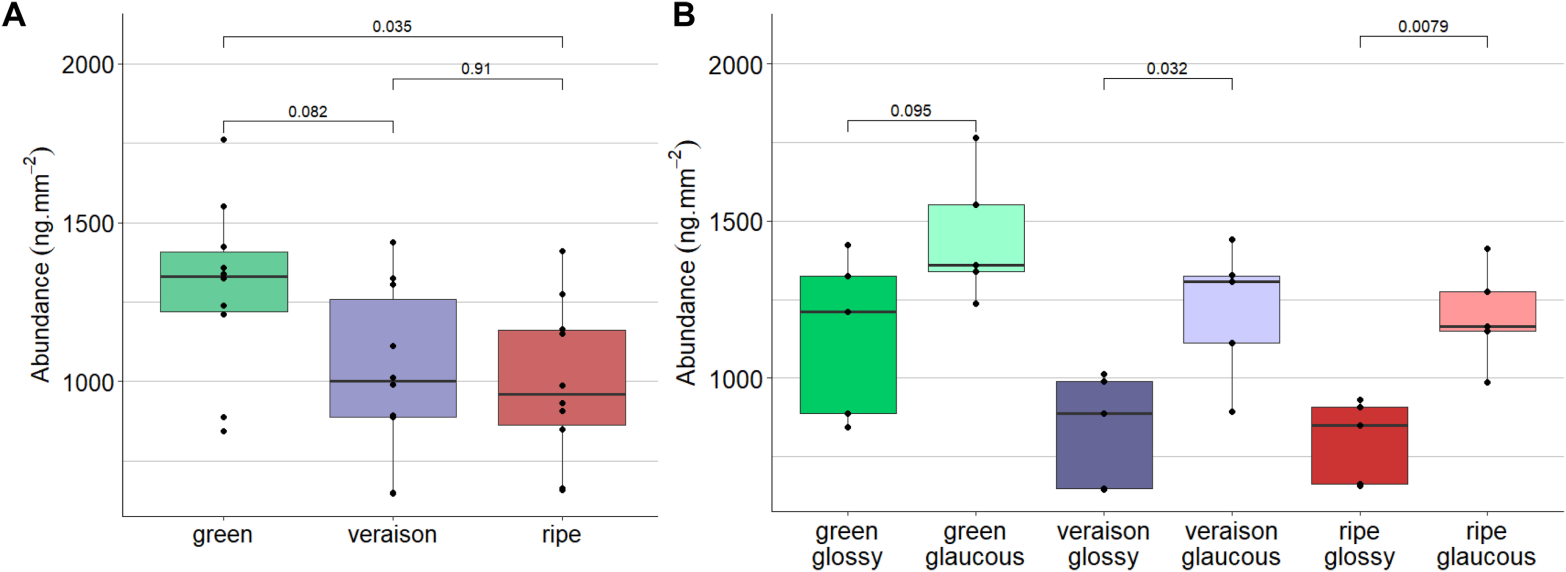
Comparison of total cuticular wax in grape berries. Determined as the sum of all compounds (A) between green, véraison, and ripe stages, and (B) between the glossy (*n*=4) and glaucous (*n*=4) phenotypes for the green, véraison, and ripe stages. Relative abundance is indicated in ng mm^–2^. Group means were compared and *P*-values between groups are indicated on the graphs.

The berry cuticular waxes of selected lines were sampled during the green, véraison, and ripe stages of berry development in the season to determine the developmental differences of the wax composition. The total wax significantly decreased by 23% between the green and ripe stages (*P*=0.035, Fig. 4). No significant differences were observed between directly successive stages, suggesting that the wax gradually decreased throughout the season. When the total wax was compared between glossy and glaucous phenotypes, there was no difference at the green stage; however, as the season progressed, the difference became more pronounced (*P*=0.032 at véraison and *P*=0.008 at ripe).

The relative abundance of single compounds was compared across the different developmental stages (Supplementary Table S3). Interestingly, it was mostly the fatty acids that showed significant differences during berry development. A total of seven fatty acids (C18:2, C20, C22, C26, C28, C30, and C32 fatty acids) as well as the total fatty acids were significantly different between different stages (Supplementary Fig. S3). The shortest (C18:2) and longest (C32) fatty acids decreased during the season, while the other fatty acids increased. The timing of this increase varied for each fatty acid: C30 increased at the start of the season, C20 and C28 constantly increased throughout the season (significantly between each stage), C26 gradually increased (significantly only between green and ripe), and C22 increased only at the end of the season. Total fatty acids also showed a significant increase towards the end of the season. Additionally, one aldehyde (C32) gradually decreased throughout the season, with a significant difference between green and ripe (*P*=0.0089; Supplementary Table S3). Total aldehydes were not significantly different between development stages (*P*=0.35 between green and véraison, *P*=0.97 between véraison and ripe, *P*=0.10 between green and ripe). However, the ratios of aldehydes to alcohols (*P*=0.0068), alkanes (*P*=0.019), and fatty acids (*P*=0.019) were significantly higher at the green stage compared with the ripe stage (Supplementary Fig. S4). For the ratio of aldehydes to alkanes and fatty acids, this was a gradual decrease (no significant difference between successive stages), whereas for the ratio of aldehydes to alcohols, this shift occurred between green and véraison (*P*=0.0068). Aldehydes, therefore, were more abundant at the onset of berry development, with a shift in abundance to alcohols, alkanes, and fatty acids as berries ripened.

### The glaucous phenotype is driven by increases in alcohols and aldehydes

**Fig. 5.**
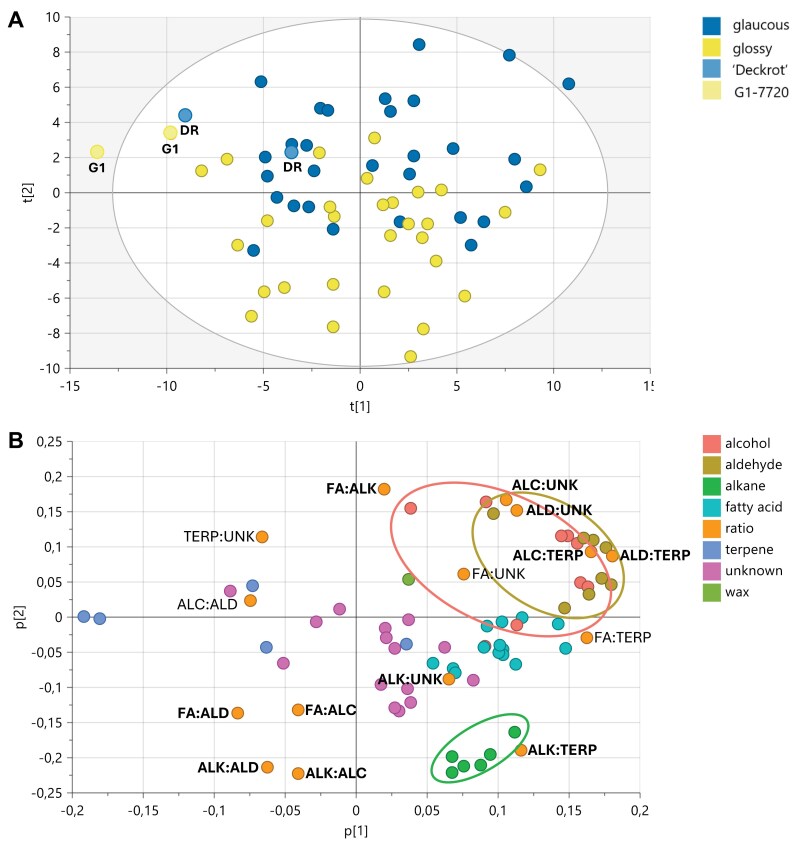
Principal component analysis (PCA) of the grape berry cuticular wax composition. Analysis performed for glossy (*n*=30) and glaucous (*n*=30) progeny and the parents, ‘Deckrot’ (glaucous) and G1-7720 (glossy). The (A) score and (B) loading plot indicate that the glaucous phenotype is associated with an increase in alcohols and aldehydes and a deficiency of alkanes (encircled in the loading plot). Compound ratios are labelled on the loading plot, with significant differences indicated in bold. ALC, alcohol; ALD, aldehyde; ALK, alkane; DR, ‘Deckrot’; FA, fatty acid; G1, G1-7720; TERP, terpenes; UNK, unknown.

The wax composition of the G1-7720 and 30 glossy offspring was compared with that of ‘Deckrot’ and 30 glaucous offspring. On average, the glaucous phenotype contained 9.84% more wax than the glossy phenotype (*P*=0.0085). PCA explained 29.4% variance on the first principal component (*x*-axis) and 17.5% on the second principal component (Fig. 5A). Although no clear clusters were observed, the waxy phenotypes were loosely separated along the second principal component. This separation was driven by a significantly higher relative abundance of alcohols (C22, C24, C25, C26, and total alcohols) and aldehydes (C24, C26, and total aldehydes) in the glaucous phenotypes (Fig. 5B). Alternatively, the glossy cohort had a significantly higher relative abundance of fatty acids (C20 and C22 fatty acids) and alkanes (C25, C27, C29, C31, and total alkanes). This effect is also observed in the compound ratios, with all ratios significantly different between glossy and glaucous, including the ratios with alcohols and aldehydes. Furthermore, regarding the variation in total aldehydes across the developmental stages (Supplementary Fig. S5), there was a significant difference between glaucous and glossy individuals (*P*=0.008 for green and véraison, and *P*=0.012 for ripe), with glaucous samples always accumulating more aldehydes than glossy samples. The total triterpenoids were 9.08% higher in the glaucous cohort (*P*=0.048), but none of the individual triterpenoids differed significantly between glaucous and glossy phenotypes.

### The composition and load of grape berry cuticular wax are dynamic and genotype specific

**Fig. 6.**
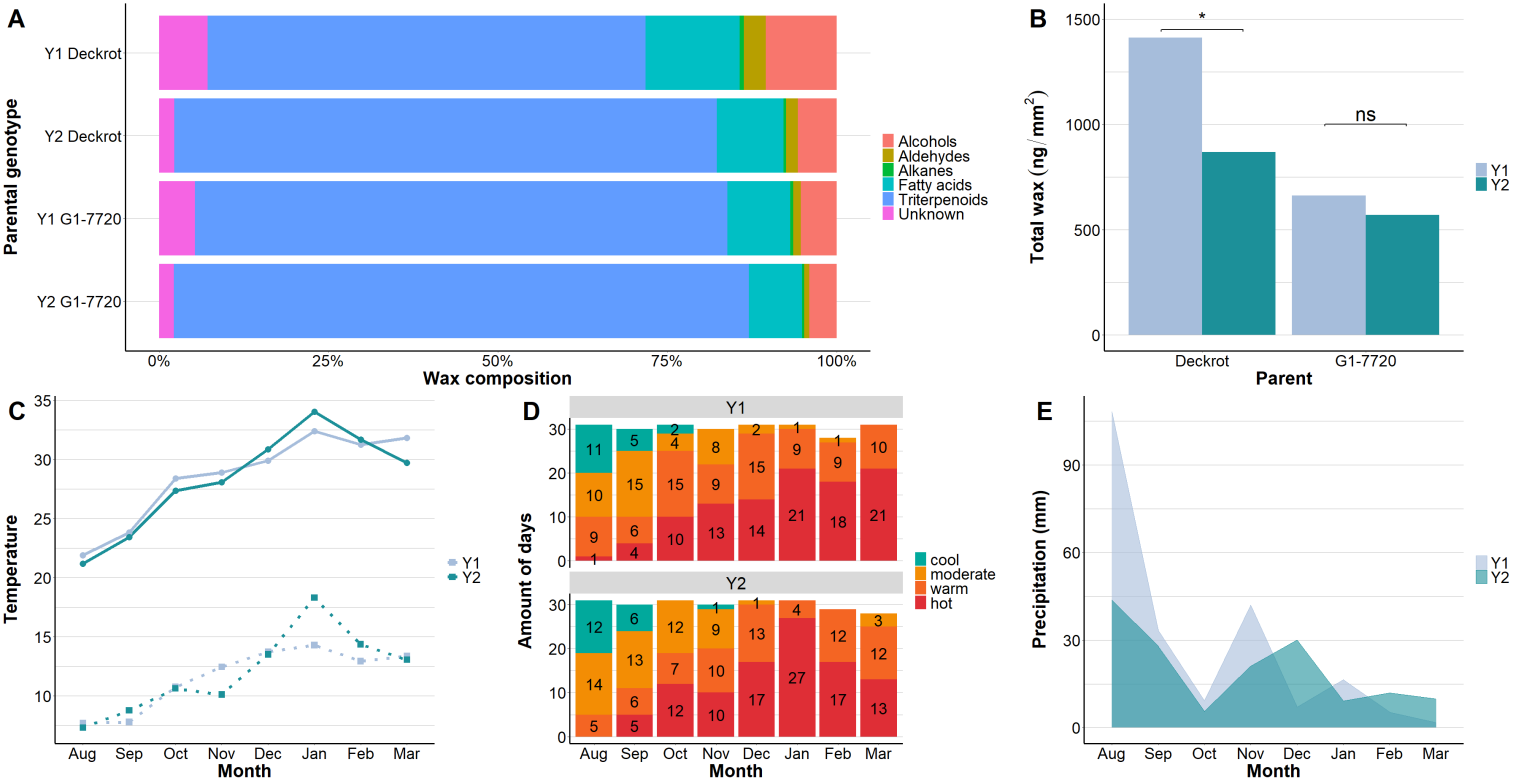
The role played by the environment on grape berry wax composition. Comparison of the grape cuticular wax of ripe berries between the parental genotypes, ‘Deckrot’ (glaucous) and G1-7720 (glossy). The composition (A) and the total wax (B) of the two parents are compared across two years (Y1 and Y2). A detailed breakdown of the compositional differences can be found in Supplementary Table S4. The climatic conditions of the two seasons (Y1 and Y2) are compared within the context of (C) minimum and maximum temperature, (D) number of cool (<20 °C), moderate (20-25 °C), warm (25-30 °C), and hot (>30 °C) days, and (E) amount of precipitation.

The wax load and composition of ripe berries of the two parents were compared across 2 years (Fig. 6A–E). For these 2 years, rainfall was similar during the flowering stage (September and October), whereas Y1 received rain mostly during fruit set (November) and not during pre-véraison (December). In Y2, precipitation was spread over fruit set and pre-véraison (November and December). The minimum and maximum temperatures were similar during the flowering stage (September and October); however, during fruit set (November), the minimum temperature was lower in Y2 than in Y1. In general, Y2 was hotter than Y1 with more warm (25–30 °C) and hot (>30 °C) days, especially during post-véraison (January). In summary, Y2 had a lower minimum temperature during fruit set, and hotter temperatures during post-véraison. The wax load was 1.58 times higher (*P*=0.0204; Supplementary Table S4) in Y1 than in Y2 for ‘Deckrot’ (glaucous). This difference in wax load in ‘Deckrot’ can be attributed to a 30% reduction of oleanolic acid, a 55% reduction in total fatty acids (especially C24, C26, and C28), and a 65% reduction in total alcohols (especially C24 and C28) (Supplementary Table S4). Although the absolute oleanolic acid was reduced in Y2, the relative contribution of oleanolic acid to the wax composition was increased in Y2 (Fig. 6A). For G1-7720 (glossy), the wax load did not significantly differ between the two years.

**Fig. 7.**
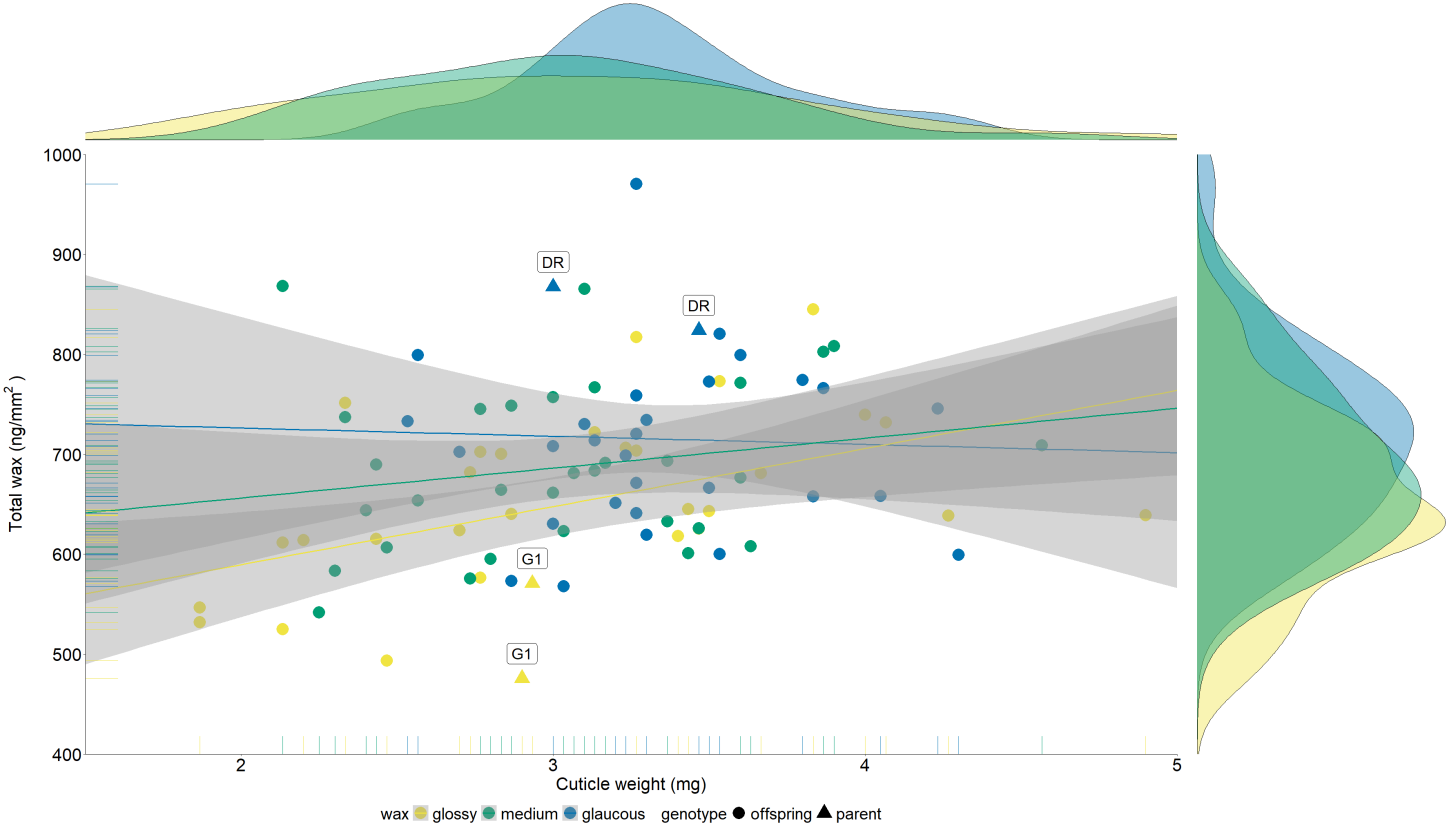
Comparison and distribution of the total wax load and cuticle weight. Data are shown for the mapping progeny (categorized as glossy, medium, or glaucous) and parental genotypes, ‘Deckrot’ (DR) and G1-7720 (G1). Trendlines and confidence intervals are indicated for each wax phenotype (glossy, medium, or glaucous).

The cuticular wax composition was compared between the two parental genotypes, ‘Deckrot’ and G1-7720 (Fig. 6A). The berry wax of glaucous ‘Deckrot’ contained more alcohols and aldehydes compared with the glossy G1-7720. More specifically, the C26-aldehyde, C32-aldehyde, C24-alcohol, and C32-alcohol were 3–4 times higher in the berry wax of ‘Deckrot’ than in the G1-7720 berry wax (Supplementary Table S4). Interestingly, the C16-fatty acid was 2.42 times higher in the ‘Deckrot’ wax. The segregation of berry cuticular wax traits was investigated in the ‘Deckrot’×G1-7720 mapping population (Fig. 7). Although the wax load showed much overlap between the wax phenotype groups, the trendlines indicate that the wax load was highest in the glaucous phenotypes, followed by the medium and glossy phenotypes. The correlation between wax load and wax phenotype groups was significant (*P*=0.00045, *R*^2^=0.35), indicating that the wax load contributes to the visual appearance of wax. The wax load ranged from 493.36 ng mm^–2^ to 970.57 ng mm^–2^ across the progeny, with the parent genotypes being near the extremes of the range (523.28 ng mm^–2^ for G1-7720 and 845.89 ng mm^–2^ for ‘Deckrot’, along the vertical axis in Fig. 7). The correlation between cuticle weight and wax phenotype groups was significant, but less so (*P*=0.047, *R*^2^=0.21). Cuticle weight is a compound measurement of the cuticle thickness and wax load, and this correlation is most probably determined by the wax load component. In the offspring, the cuticle weight ranged from 1.87 mg to 5.27 mg, whilst parents showed less extreme phenotypes (2.92 mg for G1-7720 and 3.23 mg for ‘Deckrot’, along the horizontal axis in Fig. 7). This suggests that the parents are most probably differentiated on wax load rather than cuticle thickness, whilst the progeny segregate for both. The variation in wax composition and load in the parents and offspring provides support for wax trait segregation and the suitability of this population for QTL analysis.

### Metabolic QTL analysis identifies several novel loci underlying berry wax accumulation

The mapping identified QTLs in the ‘Deckrot’×G1-7720 population for the wax compound concentrations on 11 of the 19 linkage groups (Table 1; Supplementary Table S5). On the ‘Deckrot’ map, significant QTLs were identified for 15 traits (C22 alcohol; C18:1-, C20-, C22-, and C24 fatty acids; α-amyrin; β-amyrin; and unknown compounds 2, 4, 5, 8, 9, 10, 11, and 13). On the G1-7720 map, significant QTLs were also identified for 15 traits (C20-, C24-, and C28 alcohols; C24 aldehyde; C20-, C22-, and C28 fatty acids; α-amyrin; β-amyrin; and unknown compounds 2, 4, 5, 7, 8, and 12). These QTLs represented 24 different QTL regions across 11 chromosomes (chromosomes 2, 3, 4, 5, 6, 7, 8, 9, 10, 17, and 18).

**Table 1.** Quantitative trait loci identified for grape berry wax compounds in the ‘Deckrot’×G1-7720 mapping population

Region	Trait	Parent	Position	No. of genes	LOD	PVE
2.1	Unknown12	G1	chr02:3550864 … 3696957	19	2.82	10
2.2	C18-oleic acid (C18:1)	DR	chr02:7257173 … 7307734	3	4.49	20.5
2.3	Unknown11	DR	chr02:7412455 … 7586935	11	3.71	17.3
2.4	Unknown8	DR	chr02:9055455 … 14934862	330	4.32	20.0
	Unknown2	DR	chr02:9086122 … 15054521	333	3.52	16.5
2.5	C28 fatty acid	G1	chr02:15524738 … 15947743	38	3.11	14.7
2.6	Unknown8	G1	chr02:17806757 … 18472407	51	3.57	14.3
3.1	Unknown5	G1	chr03:5023752 … 6074070	109	3.17	13
3.2	C20 fatty acid	DR	chr03:6519604 … 6907296	34	3.73	12.1
3.3	C28 alcohol	G1	chr03:9430388 … 10836383	88	4.28	19.7
3.4	Unknown13	DR	chr03:10913877 … 12342861	90	4.98	14.0
4.1	C22 alcohol	DR	chr04:18568378 … 19262413	85	4.29	19.7
4.2	C22 alcohol	G1	chr04:19361911 … 20108478	84	7.82	33.4
	C24 aldehyde	G1	chr04:19681941 … 20171356	57	4.38	20.6
	C24 alcohol	G1	chr04:19681941 … 20171356	57	4.44	21.1
5.1	Unknown7	G1	chr05:1437964 … 2723418	142	9.29	37.8
	C20 fatty acid	G1	chr05:2217503 … 2823899	67	7.15	30.6
	C22 fatty acid	G1	chr05:2217503 … 2823899	67	6.03	26.6
5.2	Unknown5	G1	chr05:2823899 … 4301838	170	3.33	13.2
	α-Amyrin	G1	chr05:2898965 … 4373666	162	4.79	21.7
	Unknown8	G1	chr05:4058168 … 4424256	34	4.42	16.8
6.1	Unknown2	G1	chr06:14193165 … 14363834	10	3.73	17.4
7.1	Unknown9	DR	chr07:25267990 … 25818799	32	3.3	15.6
7.2	Unknown5	DR	chr07:26046906 … 26253561	15	4.43	20.3
8.1	Unknown13	DR	chr08:13202273 … 13401437	16	10.65	32.7
9.1	Unknown4	G1	chr09:16910956 … 21710583	347	7.46	31.7
	β-Amyrin	DR	chr09:15037003 … 20056555^*a*^	563	3.4	16.0
	Unknown4	DR	chr09:17961463 … 20056555^*a*^	372	6.72	29.1
	α-Amyrin	DR	chr09:17961463 … 20056555^*a*^	372	12.57	47.4
	Unknown10	DR	chr09:17961463 … 20056555^*a*^	372	8.37	34.8
10.1	Unknown12	G1	chr10:11409080 … 11726790	14	6.82	26.9
17.1	C20 fatty acid	DR	chr17:909693^*a*^...953730	118	3.92	14.5
	C24 fatty acid	DR	chr17:909693^*a*^...953730	118	3.36	16.4
17.2	C22 fatty acid	DR	chr17:5903974 … 6202154	33	5.5	24.7
18.1	β-Amyrin	G1	chr18:10312345 … 10364075	8	3.12	12.3
18.2	Unknown5	G1	chr18:29348169 … 30223896	70	4.51	18.7

For each QTL region, the respective trait, the parental map in which it was mapped, associated genomic regions based on the ‘PN40024.v3’ reference genome, number of annotated genes in this genomic region, the LOD, and percent variation explained (PVE) are indicated. DR, ‘Deckrot, G1, G1-7720

^*a*^ QTL region mapped to the first/last marker on map, position indicates the genomic position of marker, but the distal end of the chromosome was used for candidate gene identification.

The annotated genes in each identified QTL region were inspected to identify putative candidate genes (Table 2). The candidate genes included the well-known wax synthesis genes *WSD1* and *CER3*. Various genes that are involved in fatty acid metabolism, such as *SAD6*, *ACO32*, *AL3F1*, and *FATB,* were also identified as possible candidate genes. Considering that 70% of the wax consists of triterpenoids, it is not surprising that a number of genes contribute to triterpenoid synthesis, such as *EOT1*, *GGPS1*, *BAMS*, and *CYP716A*. The QTL region on chromosome 9 was of particular interest as this region showed a very high LOD score of 12.57 and explained 47.4% of the variance for α-amyrin. This region contained 16 genes that were annotated as β-amyrin synthases (*BAMS*, *BAMS1*, or *BAMS2*).

**Table 2.** Putative candidate genes identified for the QTL regions for grape berry waxes in the ‘Deckrot’×G1-7720 mapping population

Region	Candidate gene(s) in QTL region	Function
2.4	*Stearoyl-[acyl-carrier-protein] 9-desaturase 6* (*SAD6*)	Fatty acid desaturation^1^
	*ABC transporter G family member 36* (*ABCG36*)	Cutin deposition, cellular detoxification^2^
	*Putative acyl-coenzyme A oxidase 3.2* (*ACO32*)	Fatty acid metabolism^3^
2.6	*Long-chain-alcohol oxidase* (*FAO4A*)	Fatty alcohol oxidase^4^
3.1	*Probable carboxylesterase 7* (*CXE7*)	Carboxylesterase present in *Cer-cqu* wax loci^5^
	*Probable carboxylesterase 1* (*CXE1*)	Carboxylesterase present in *Cer-cqu* wax loci^5^
	*Probable carboxylesterase 2* (*CXE2*)	Carboxylesterase present in *Cer-cqu* wax loci^5^
	*O-acyltransferase WSD1* (*WSD1*)	Wax ester synthesis^6^
4.1	*Abscisic acid 8'-hydroxylase* (*ABAH3*)	Abscisic acid catabolism^7^
4.2	*ABC transporter G family member 24* (*ABC24G*)^*a*^	ABC transporter^8^
	*Protein EXPRESSION OF TERPENOIDS 1* (*EOT1*)	Transcription factor for terpene biosynthesis^9^
	*Aldehyde dehydrogenase family 3 member F1* (*AL3F1*)	Fatty acid degradation^10^
5.1	*Diacylglycerol O-acyltransferase 3* (*DGAT3*)^*b*^	Fatty acid composition^11^
5.2	*Geranylgeranyl pyrophosphate synthase large subunit 1* (*GGPS1*)^*c*^	Terpene biosynthesis^12^
	*Acyl carrier protein 1* (*ACP1*)^*c*^	Cofactor in fatty acid biosynthesis^13^
6.1	*Non-specific lipid transfer protein GPI-anchored 2* (*LTPG2*)	Cuticular wax export^14^
9.1	Probable *WRKY transcription factor 40* (*WRKY40*)^*d,e*^	WRKY transcription factors involved in wax biosynthesis^15^
	*Very-long-chain aldehyde decarbonylase* (*CER3*)	Very long chain alkane synthesis^16^
	*Homeobox-leucine zipper protein ANTHOCYANINLESS 2* (*ANL2*)	Transcription factor regulating cutin biosynthesis^17^
	*Beta-amyrin synthase* (*BAMS*)^*d*^	Triterpenoid synthesis^18^
	*Beta-amyrin synthase 1* (*BAMS1*)^*d*^	Triterpenoid synthesis^18^
	*Beta-amyrin synthase 2* (*BAMS2*)^*d*^	Triterpenoid synthesis^18^
	*Beta-amyrin 28-monooxygenase* (*C716A52*)^*d*^	Triterpenoid biosynthesis^19^
17.1	*Beta-hydroxysteroid dehydrogenase* (*HSD6*, *HSD4A*, *HSD1B*)	Lipid homeostasis^20^
	*Palmitoyl-acyl carrier protein thioesterase* (*FATB*)	Fatty acid synthesis^21^
18.2	*Beta-amyrin 28-monooxygenase* (*C7A12*, *C7A15*, *C7A17*)	Triterpenoid biosynthesis^22^

Candidate genes are listed with their gene function.

1, Klinkenberg *et al*. (2014); 2, Elejalde-Palmett *et al*. (2021); 3, Wang *et al*. (2017); 4, Yang *et al*. (2022); 5, Schneider *et al*. (2016); 6, Li *et al*. (2008); 7, Qi *et al*., 1998); 8, Li *et al*. (2024); 9, D’Esposito *et al*. (2021); 10, Li *et al*. (2020); 11, Turchetto-Zolet *et al*. (2016); Zhao *et al*. (2021); 12, Xi *et al*. (2016); 13, Branen *et al*. (2001); 14, Kim *et al*. (2012); 15, More *et al*. (2019); 16, Rowland *et al*. (2007); 17, Nadakuduti *et al*. (2012); 18, Wu *et al*. (2019); 19, Suzuki *et al*. (2018); 20, Zhang *et al*. (2016); 21, Kunst and Samuels (2003); Lewandowska *et al*. (2020); 22, Fukushima *et al*. (2011).

^a^Associated only with C22_alcohol in this QTL region; ^b^ associated only with C20_fatty acid and C22_fatty acid in this QTL region; ^c^ associated only with unknown5 and α-amyrin in this QTL region; ^d^ associated only with a QTL region that was mapped in ‘Deckrot’; ^e^ associated only with unknown4 in this QTL region.

Full gene IDs are indicated in Supplementary Table S5.

### Expression analysis identifies *VvTTPS12* as a functional triterpene synthase

Nine candidate grapevine triterpene synthase geness (*VvTTPS1*–*10*) have been previously identified in the V0 annotation (Pensec *et al*., 2016; Supplementary Table S6). *VvTTPS3* was originally identified and named as two separate genes, but these were subsequently found to be the same gene. Here, three additional putative terpene synthase genes (*VvTTPS11*–*13*) and three cycloartenol synthase geness (*VvCAS1_1*–*3*) were identified using a homology search of the VCost.v3 annotation. The grapevine terpene synthase genes were annotated as *β-amyrin synthases*. The *β-amyrin synthase* genes were located in two clusters, one on chromosome 9 (which was also identified in the QTL analysis) and one on chromosome 10.

**Fig. 8.**
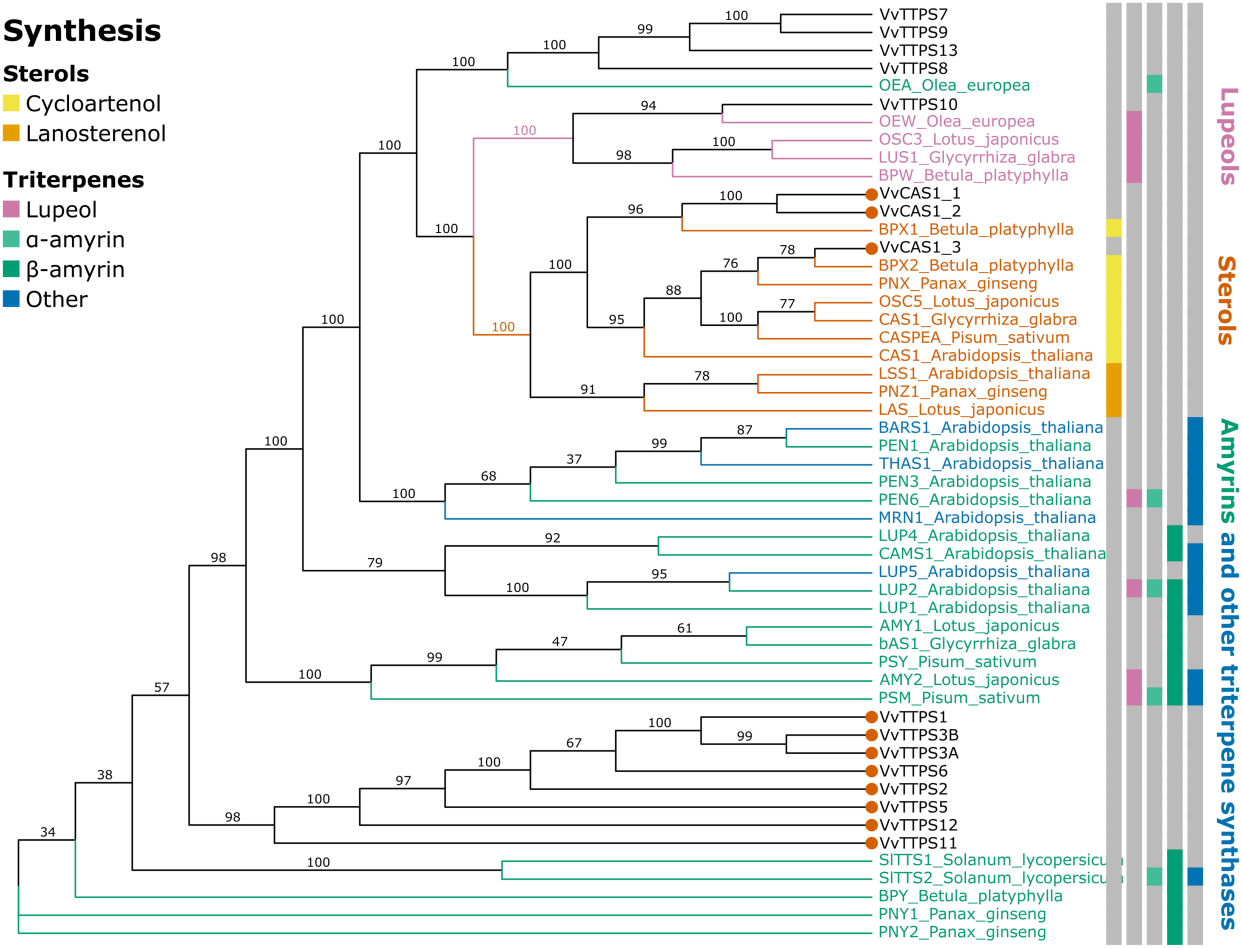
Phylogenetic analysis of grapevine triterpene synthases. The tree was constructed using amino acid sequences from 12 putative grapevine triterpene synthases (VvTTPS1–13), three putative grapevine cycloartenol synthases (VvCAS1_1–3), as well as other characterized plant terpene synthases (Supplementary Table S7). The clades are coloured according to the known synthesis function, with lupeol synthases in pink, sterol synthases in orange, amyrin synthases in green, and other triterpene synthases in blue. The triterpene synthases in QTL region 9.1 are indicated with a filled orange circle.

Phylogenetic analysis of the grapevine triterpene synthases and other plant triterpene synthases showed that the terpene synthases were separated into the lupeol, sterol, and amyrin synthases (Fig. 8; Supplementary Table S7). In agreement with the gene annotation, the *VvCAS1* genes were grouped with the sterol synthases. The group of triterpene synthases on chromosome 10 was grouped in a clade of its own. The β-amyrin synthases identified in the QTL region 9.1 shared homology with other amyrin synthases (either α-, β-, or multifunctional amyrin synthases).

**Fig. 9.**
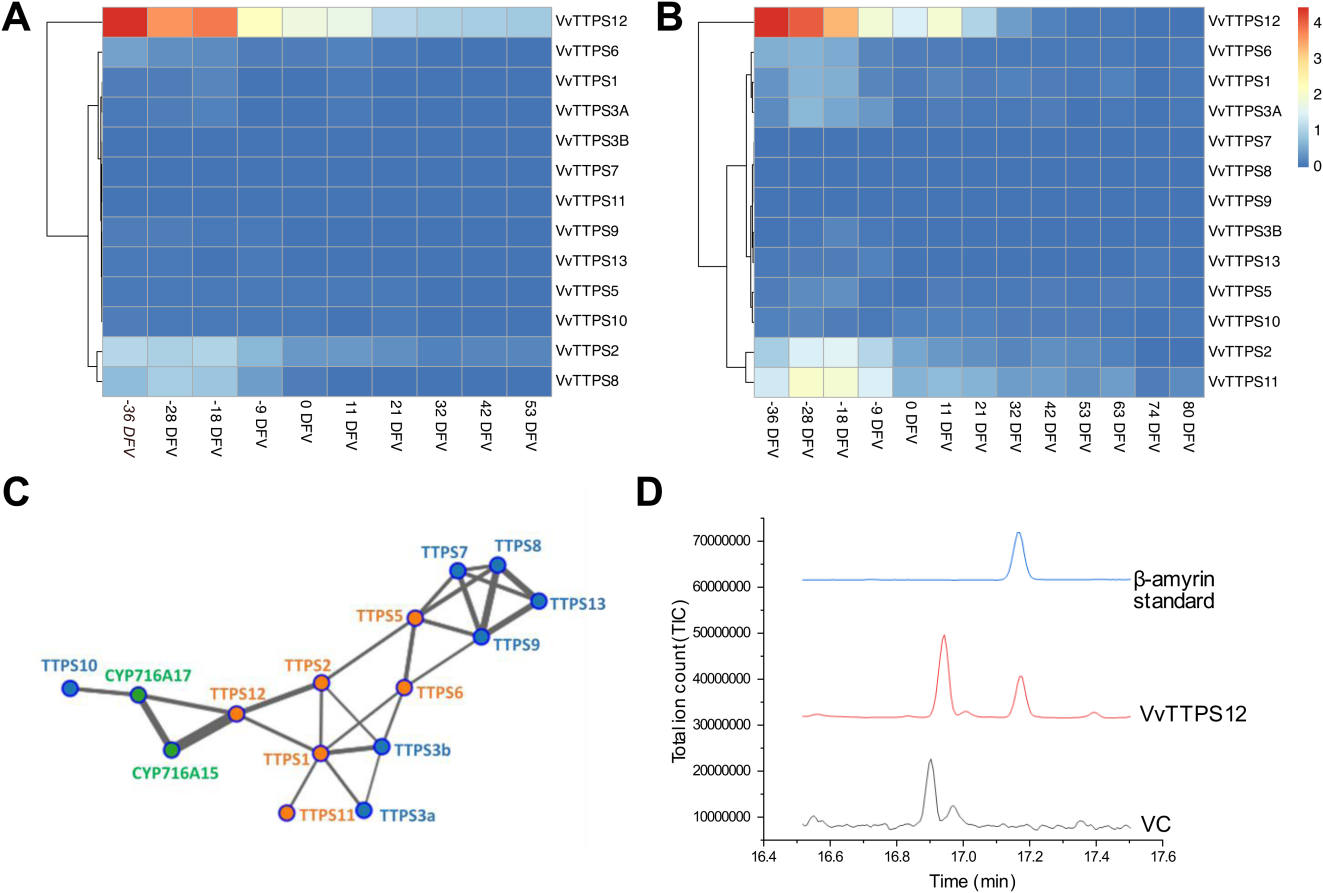
Functional characterization of grapevine candidate triterpene synthases. The expression of the grapevine terpene synthases during berry development in (A) Pinot Noir and (B) Cabernet Sauvignon. The gene expression was plotted against days from véraison (DFV). (C) Co-expression analysis revealed the co-expression network of the grapevine genes coding for CYP716A enzymes (*CYP716A15* and *CYP716A17* in green) and triterpene synthase enzymes, with *VvTTPS12* displaying direct and strong co-expression with both *CYP716A* genes. The *VvTTPS* genes located in the QTL region 9.1 are shown in orange, and other *VvTTPS* genes are in blue. The width of the line indicates the amount of co-expression between two genes. (D) Heterologous expression of *VvTTPS12* in yeast results in the production of β-amyrin. Chromatograms show an authentic standard (β-amyrin standard), an extract from yeast harbouring a vector expressing the VvTTPS12 coding sequence (*VvTTPS12*), and an extract from a yeast with an empty vector control (VC).

The expression profiles of the grapevine triterpene synthases were further investigated using expression data from public databases (Fig. 9A–C). The majority of the grapevine triterpene synthases were not expressed in the berry. The newly identified *VvTTPS12* was highly expressed at the start of berry development in both ‘Pinot Noir’ and ‘Cabernet Sauvignon’, and then tapered down from véraison. Cultivar differences were observed, with *VvTTPS2*, -*6*, and -*8* expressed at low levels in ‘Pinot Noir’, whereas *VvTTPS1*, -*2*, -*6*, and -*11* were expressed at low levels in ‘Cabernet Sauvignon’ (Fig. 9A, B). The functionally characterized grapevine genes *CYP716A15* and *CYP716A17*, which code for enzymes involved in the oxidation of triterpenoid amyrins to triterpenoic acids (Fukushima *et al.*, 2011), were used as baits in a co-expression analysis, to identify potential functional amyrin synthases. Both *CYP716A15* and *CYP716A17* were strongly co-expressed with *VvTTPS12*, suggesting a functional role for *VvTTPS12* in oleanolic acid formation (Fig. 9C).

Functional characterization of the putative β-amyrin synthase *VvTTPS12* was performed by heterologous expression in yeast. An engineered *S. cerevisiae* strain (CEN.PK2-1C rox1::P_GAL1_-tHMGR P_GAL10_-ERG13 P_ERG7Δ_::P_CTR3_) with a galactose-inducible enhanced flux of the mevalonate pathway towards the triterpenoid precursor squalene and a copper-repressible sterol biosynthetic pathway was used. Expression of *VvTTPS12* under the control of galactose-inducible promoter GAL1 led to a production of 3.37 mg l^–1^ β-amyrin (Fig. 9D).

## Discussion

### Wax composition and deposition are affected by grape berry development

In this study, berry cuticular wax (intra- and epicuticular combined) consisted predominantly of the triterpenoid, oleanolic acid, as well as fatty acids, alcohols, aldehydes, and trace amounts of alkanes (Fig. 3), and, during berry development, wax composition shifted from aldehydes to fatty acids (Supplementary Figs S3, S4). In the wax biosynthesis pathway, C16–C18 fatty acids are elongated to C20–C32 fatty acids, known as VLCFAs, which are converted to wax esters and alkanes to form wax. The decrease during berry development in the ratio of aldehydes to other compounds (alkanes, alcohols, and fatty acids) suggests an initial accumulation of aldehydes. However, the low presence of alkanes in the grape berry wax (Fig. 3) indicates that very few aldehydes were subsequently converted to alkanes via the alkane-forming pathway. Simultaneously, more VLCFAs were converted to alcohols (via the alcohol-forming pathway), reducing the ratio of aldehydes to alcohols. However, the increase of VLCFAs during the season suggests an overaccumulation of substrate which could not be readily converted. This highlights an important role for fatty acid synthesis and chain elongation in grape berry wax development. In the QTL analysis of this study, several genes involved with fatty acid biosynthesis were identified. These include *stearoyl-acyl carrier protein desaturase 6* (*SAD6*), *diacylglycerol O-acyltransferase 3* (*DGAT3*), and *palmitoyl-acyl carrier protein thioesterase* (*FATB*) which increase C16–C18 fatty acids, as well as *aldehyde dehydrogenase family 3 F1* (*AL3F1*), *fatty alcohol oxidase 4* (*FAO4A*), and *acyl-CoA oxidase* (*ACO32*) involved with the degradation of fatty acids. In particular, the identification of two candidate *FATB* genes (Vitvi17g01339 and Vitvi17g01340) on chromosome 17 is interesting, as this region has been associated with variation in C20 and C24 fatty acid content. Down-regulation of *FATB* in navel oranges (*Citrus sinensis*) was associated with a decrease of wax aliphatic compounds (Wan *et al.*, 2020). These results present interesting candidate genes to investigate further for their role in berry grape wax development.

In addition to changes in wax composition, the wax deposition decreased substantially between green and ripe berries (Fig. 4). In many fruits, such as apple, pear, blueberries, and mango, wax deposition increases throughout fruit development, but in grape and sweet cherry the cuticular waxes are deposited early in fruit development and subsequently decline (Trivedi *et al.*, 2019). This reduction in wax deposition, coupled with the rapid expansion of the fruit during ripening, stretches the cuticular wax layer and further decreases the total wax per surface area (Lai *et al.*, 2016; Yan *et al*., 2024). This adds strain to the cuticular membrane, making the fruit more susceptible to microcracking. Microcracking impairs the barrier properties which increase the susceptibility to bunch rot and water uptake, which can lead to berry cracking. In grapes, the reduction in wax deposition occurs around véraison, with post-véraison berries showing increased microcracking (Becker and Knoche, 2012).

The decrease in total wax observed in this study is largely attributed to a decrease in oleanolic acid. A decrease in oleanolic acid during berry development has been reported for various grape cultivars (Pensec *et al.*, 2014; Rid *et al.*, 2018; Arand *et al.*, 2021). In sweet cherry (*Prunus avium*), two triterpenoids, ursolic acid and oleanolic acid, decreased throughout fruit development (Peschel *et al.*, 2007). The waxes of these fruit predominantly consist of triterpenoids, unlike the surface waxes of other fruits such as apples (*Malus domestica*), pears (*Pyrus bretchneideri*), and mangoes (*Mangifera indica*) which predominantly consist of aliphatic compounds, such as alkanes and alcohols (Yin *et al.*, 2011; Chai *et al.*, 2020; Wu *et al.*, 2023). This indicates that triterpenoid synthesis is paramount for grape berry wax synthesis. Interestingly, the fruit surface wax of blueberries (*Vaccinium corymbosum* and *V. ashei*) also consists mostly of triterpenoids, but the triterpenoid acids (such as oleanolic and ursolic acid) increased throughout fruit development, whilst their triterpene precursors (such as α- and β-amyrin) decreased (Chu *et al.*, 2018). A recent study speculated that the availability of these precursors is the rate-limiting step in triterpene synthesis of blueberry (*V. corymbosum*) wax (Yan *et al*., 2024). This suggests different regulatory mechanisms of triterpenoid synthesis in grape and blueberry, despite similar wax compositions.

### Diversity of grape berry cuticular waxes is genetically regulated

Glaucous individuals in this mapping population had on average 10% more wax than the glossy individuals (Fig. 7). However, the glaucous phenotype was also associated with an increased alcohol and aldehyde composition compared with the glossy individuals, in agreement with the findings of Yamamura and Naito (1983). They suggested that the visual appearance of the grape waxy bloom was to some extent related to the amount of soft wax (which includes the aliphatics), but did not identify any specific compounds. The observed increase in alcohols and aldehydes suggests an up-regulation of both alcohol- and alkane-forming pathways of the aliphatic wax biosynthesis pathway in the glaucous phenotype. Alcohols are formed by the reduction of VLCFAs by a fatty acyl-CoA reductase, CER4 (Rowland *et al.*, 2006). Dimopoulos *et al*. (2020) investigated grape *CER4* homologues; however, these homologues were not expressed when the alcohol content increased in grape berries. None of these homologues, nor other *CER4*-like genes, was identified in this QTL study. Therefore, the gene responsible for alcohol synthesis remains unidentified in grapevine. Alternatively, VLCFAs can be reduced by CER1 and CER3 to form aldehydes (Bourdenx *et al.*, 2011; Bernard *et al.*, 2012). From the QTL results, two putative *CER3* candidate genes (Vitvi09g01887 and Vitvi09g01889) were identified among the QTLs on chromosome 9 (Table 2). However, it has been reported that these candidate genes were down-regulated when aliphatic wax compounds increased (Dimopoulos *et al.*, 2020), suggesting that the functional *CER1* and *CER3* still need to be identified in grapevine. Interestingly, one of these *CER3*-like genes (Vitvi09g01889) was down-regulated by water stress (Dimopoulos *et al.*, 2020). The elusiveness of these wax synthesis genes in the presence of homologues indicates that wax synthesis pathways are species specific and require further investigation in grapevine.

Genetic characterization of the aliphatic wax biosynthesis, regulation, and transport is limited in grapevine. This is the first study that has characterized the metabolic composition of the grape berry wax in a mapping population segregating for the glossy and glaucous wax phenotypes. The glaucous phenotype of wheat (*Triticum aestivum*) and barley (*Hordeum vulgare*) plants has been linked to a metabolic cluster comprising three genes, called *W1* in wheat and *Cer-cqu* in barley (Hen-Avivi *et al.*, 2016; Schneider *et al.*, 2016). One of these genes encodes a carboxylesterase which is responsible for the hydrolysis of long-chain fatty acid esters, producing a carboxylic acid and an alcohol. Various carboxylesterase candidates have been identified in the QTL analysis of this study and could possibly contribute to the accumulation of alcohols seen in the glaucous phenotype of grape berries. In stem waxes of *Arabidopsis thaliana*, the wax synthase/diacylglycerol acyltransferase (WSD1) enzyme is responsible for synthesizing wax esters from alcohols in the alcohol-forming pathway (Li *et al.*, 2008). In this study, a candidate gene for *WSD1* (Vitvi03g00527) was identified on chromosome 3 which could contribute to accumulation of alcohols, if these are not readily converted to wax esters. Dimopoulos *et al*. (2020) found that the expression of three *WSD1*-like homologues (on chromosome 15) was consistent with changes in the wax composition during grape berry development. A functional *WSD1* for grapevine remains to be established, and the *WSD1* candidate in this study presents an interesting candidate for grape wax synthesis.

After being synthesized in the endoplasmic reticulum, cuticular lipids have to be transported through the plasma membrane and cell wall to the extracellular surface. This export is carried out by the plasma-bound ABC transporter of the ABCG subfamily (Pighin *et al.*, 2004). ABC transporters of this subfamily usually function as heterodimers, and two functional transporters, ABCG11 and ABCG12, have been identified as necessary for wax export. Loss-of-function mutants of these transporters result in a significant reduction of wax load and wax compounds (Rashotte *et al.*, 2001; Pighin *et al.*, 2004). It is suggested that additional ABCG transporters are involved in cuticular wax export, as the double mutant still had half of the wax load of the wild type. An ABCG candidate gene, *ABC24G* (Vitvi04g01372), has been associated with alcohol and aldehyde content (Table 1). *ABC24G* has not been directly linked with wax export, but it was down-regulated in the leaves of autotetraploid sour jujube (*Ziziphus jujuba*), which had a higher wax content than diploids (Li *et al.*, 2024), suggesting a possible link with wax accumulation. In this study, QTL detection was based on metabolic data from a single year, which may have resulted in false positives, or the identification of unstable minor QTLs. To ensure reliability, further studies are required to confirm these findings. Candidate genes were selected on the basis of supporting evidence from the literature and present a valuable contribution to understanding genetic regulation of wax composition in grapevine.

While a number of QTLs for wax formation were identified, the potential impact of genotypic response to abiotic factors on wax deposition was observed. The total wax of ‘Deckrot’ decreased by 37% in Y2, whereas G1-7720 showed no significant difference between Y1 and Y2 (Fig. 6B). As most waxes are deposited before véraison, it is likely that abiotic factors during fruit set and up to véraison had the greatest impact on wax deposition. In this study, seasonal differences in abiotic factors were attributed to a cooler and wetter season in Y2, whereas Y1 had several high temperature days in the pre-véraison period (Fig. 6C–E). Low temperatures have been observed to impact cuticular wax accumulation in other species, but findings are contradictory and still poorly understood (Carvajal *et al.*, 2021; Rahman *et al.*, 2021; Zhu *et al.*, 2022). Rain can erode cuticular waxes from leaves (Baker and Hunt, 1986). In some of these species, leaf waxes initially decrease, but are able to recover, whereas in other species leaf waxes continue to decrease during precipitation. The erosion effect of rain on grape or other fruit surface waxes has not been studied. In leaf cuticular waxes of the medicinal plant *Aspidosperma pyrifolium*, drier years such as Y1 can result in increased wax load (Medeiros *et al.*, 2017; Slot *et al.*, 2021). In blueberries (*V. corymbosum*), total triterpenoids, and in particular oleanolic acid, were negatively correlated to water loss (Yan and Castellarin, 2022). Heatwaves have been shown to increase oleanolic acid and β-amyrin in grapes (VanderWeide *et al.*, 2022). Triterpenoids are hydrophobic and form a nanocomposite with cutin to improve the mechanical toughness of the cuticular membrane, aiding in protection against biotic and abiotic stresses (Tsubaki *et al.*, 2013). About half of the difference in total wax in ‘Deckrot’ between the two years in this study could be attributed to the difference in oleanolic acid concentration. This study observed that two genotypes potentially have different capacities to respond to abiotic factors. Compared with G1-7720, ‘Deckrot’ showed more plasticity and was able to adapt the wax load accordingly. Similarly, Rahman *et al*. (2021) observed that waxier genotypes of *A*. *thaliana* were more able to accumulate wax under cold stress. The lack of QTL detection across multiple years prevents the detection of QTLs that are expressed under varying environmental conditions. Identifying and understanding the genetic mechanisms underlying regulation of wax formation should help to identify and develop genotypes more resilient to variation in abiotic (stress) factors.

### Cuticular wax plays an intricate role in grapevine plant pathogen defence

The cuticular waxes are the first barrier against external stresses, such as pathogens. Here the glaucous parent ‘Deckrot’, which contained more visible wax, was less susceptible and did not become infected with *B*. *cinerea* (Fig. 2). In contrast, the glossy G1-7720 parent, with reduced visual wax phenotype, became severely infected. Greater wax content in grape berries has been previously linked to decreased susceptibility to *B*. *cinerea* (Marois *et al.*, 1986; Rosenquist and Morrison, 1988; Percival *et al.*, 1993; Commenil *et al.*, 1997; Gabler *et al.*, 2005). Therefore, it was thought that genotypic variation among cultivars with respect to the amount of wax potentially results in differential defence responses against fungal pathogens, such as *B*. *cinerea*. However, progeny of contrasting visual wax phenotypes did not exhibit a consistent pattern of susceptibility. Notably, the glossy offspring DG055, selected for reduced visible wax, remained free from visible *B*. *cinerea* infections—in both mycelial growth and necrotic lesions, which often precede mycelial growth (Weiller *et al*., 2021; Supplementary Fig. S2). The observed variation in susceptibility in the small number of offspring tested during infection suggested that the visual observation of the waxy phenotype was insufficient to draw conclusions about the role of cuticular waxes in fungal susceptibility.

The removal of the cuticular waxes increased susceptibility to *B*. *cinerea* infection significantly (Fig. 2). Although chloroform has been used to study fungal infections on dewaxed fruit surfaces (Oh *et al.*, 1999; Chen *et al.*, 2014), it is possible that the treatment could have altered additional properties of the grape berry, such as exudation of sugars, which could further contribute to increased fungal infection (Marois *et al.*, 1986). However, these results indicate that the cuticular waxes have an effect on the interactions with the fungal pathogen. These plant–pathogen interactions are intricate, as cuticular waxes have been associated with reduced fungal growth (Li *et al.*, 2014; Tang *et al.*, 2017; Zhu *et al.*, 2020), but also with stimulatory effects on fungal infection, by inducing germination and differentiation during the pre-penetration processes (Hansjakob *et al.*, 2011; Zhu *et al.*, 2020). These interactions are inherently dependent on the type (and even strain) of pathogen, as well as the co-evolutionary dynamics between the pathogen and plant defence mechanisms. However, the variation in wax composition during grape berry development, as well as between different wax phenotypes as observed in this study, suggests that wax composition variation could also play a role in this interaction. Interestingly, on the basis of wax composition, individuals from the *B*. *cinerea* susceptibility assay clustered into three groups, two glossy and one glaucous (Supplementary Fig. S6). The second glossy group, which included the less susceptible glossy genotypes (DG055 and DG223), was associated with increased alkanes. All alkanes were increased in these two glossy individuals, and even more so than in the glaucous individuals, suggesting that alkanes could potentially be involved with susceptibility to *B*. *cinerea*. However, this was tested in a small number of samples and warrants further investigation to fully elucidate the effect of the wax composition on the defence response.

### Triterpene synthases are pivotal to grape berry wax formation

Oleanolic acid is a pentacyclic triterpenoid which contributes to 50–80% to the total berry surface wax in grapevine (Fig. 3; Radler, 1965; Commenil *et al*., 1997; Rid *et al*., 2018). The significant proportion of triterpenoids in grape berry wax underscores the crucial role of triterpenoid biosynthesis in grape berry wax formation. Triterpene and sterol synthases are oxidosqualene cyclases (OSCs) which cyclize a common precursor, 2,3-oxidosqualene. Higher plants contain several OSCs which have arisen through gene duplication. This was also evident here with the identification of 17 putatively functional OSC genes, including 12 triterpene synthases and three sterol synthases (Supplementary Table S6). Tandem repeats of the OSC gene family often occur in plant genomes (Cárdenas *et al.*, 2019) and explain the alignment of the phylogenetic groupings with the chromosomal location of the triterpene synthesis genes, with two clusters of triterpene synthases and a single lupeol synthase (Fig. 8). The large size of this gene family, together with the presence of multiple gene copies, makes it challenging to identify the functional genes responsible for specific compounds. In the case of oleanolic acid, 2,3-oxidosqualene is cyclized to the β-amyrin scaffold by the OSC β-amyrin synthase. In some cases, β-amyrin can also be formed by mixed-function OSCs that make a combination of triterpenoid scaffolds. Multiple β-amyrin synthases have been functionally characterized in several plants (Kushiro *et al.*, 1998; Morita *et al.*, 2000; Iturbe-Ormaetxe *et al.*, 2003), but not yet in grapevine. Although both clusters of triterpene synthases were annotated as β-amyrin synthases, the cluster on chromosome 10 grouped with a known single function α-amyrin synthase in olive, whereas the cluster on chromosome 9 grouped with several multifunctional α- and β-amyrin synthases. This suggested that one of the annotated β-amyrin synthases on chromosome 9 was more likely to synthesize β-amyrin. After β-amyrin is synthesized, it is oxidized by a single member of the CYP85 family of P450s. The CYP716A12 enzyme was first identified in *Medicago truncatula* (Carelli *et al.*, 2011), and subsequently two functional homologues (CYP716A15 and CYP716A17) were identified in grapevine (Fukushima *et al.*, 2011). Co-expression analyses with these homologues indicated that *VvTTPS12* was the only triterpene synthase gene that is co-expressed with the CYP85 enzymes necessary for oleanolic acid formation, suggesting that *VvTTPS12* encodes a functional β-amyrin synthase. Furthermore, the expression profile of *VvTTPS12* in the berry aligns with the decrease in oleanolic acid that has been observed during grape berry wax development (Fig. 9A, B; Pensec *et al.*, 2014; Rid *et al.*, 2018; Arand *et al.*, 2021). *VvTTPS12* is highly expressed at the start of berry development and decreases just before véraison. Lastly, heterologous expression of *VvTTPS12* successfully produced β-amyrin (Fig. 9D). Therefore, although the QTL was identified in a single year within a limited population size, the high LOD score of this QTL, together with the additional gene expression and functional analysis, provides compelling evidence linking *VvTTPS12* to oleanolic acid accumulation in grapevine berry wax.

### Conclusions

Cuticular waxes are interesting as they are the first barrier of the plant to the external environment. This study characterized the composition of grape berry cuticular wax of ‘Deckrot’ and G1-7720, as well as of the progeny from the cross of these two genotypes. The variation in compositional differences indicates that wax composition is dynamic and genotype specific. To date, very few genes have been identified that are involved in the cuticular wax formation in grape berries. This study is the first metabolic QTL study of grapevine berry wax, and various candidate genes were identified for the first time in grapevine. Furthermore, it was successfully shown that *VvTTPS12* is a functional β-amyrin synthase, whose expression profile matches the wax development pattern. This suggests that *VvTTPS12* most probably plays a major role in triterpenoid synthesis of grape berry cuticular wax. These insights provide a better understanding of the grape berry cuticular wax composition and regulation.

## Supplementary data

The following supplementary data are available at *JXB* online.

Table S1. Summary and description of the samples used in this study.

Table S2. Summary of the compounds in the grape berry cuticular wax as measured through GC-MS.

Table S3. Statistical analysis results of significant differences in compound concentrations between different developmental stages.

Table S4. Statistical analysis results of significant differences in compound concentrations between Y1 and Y2 in the parent ‘Deckrot’.

Table S5. List of annotated genes (and putative candidate genes) in significant QTL regions.

Table S6. List of candidate grapevine triterpene synthase (*VvTTPS*) genes.

Table S7. List of functionally characterized plant terpene synthase genes used for phylogenetic analysis.

Fig. S1. Comparison of mycelial scores between berries with intact wax and with removed wax.

Fig. S2. *B*. *cinerea* infections in parents and select offspring over 8 d.

Fig. S3. Comparison of the relative abundance of various fatty acids in grape berry cuticular waxes.

Fig. S4. Comparison of aldehyde ratios across different developmental stages.

Fig. S5. Comparison of total aldehydes between glossy and glaucous phenotypes across different developmental stages.

Fig. S6. Compositional differences in cuticular wax of grape berries and *Botrytis cinerea* susceptibility.

eraf119_suppl_Supplementary_Tables_S1-S7_Figures_S1-S6

eraf119_suppl_Supplementary_File_2

## Data Availability

All data supporting the findings of this study are available within the paper and within its supplementary data published online. Any data not shown are available from the corresponding author upon request.

## Abbreviations:

ABC: ATP-binding cassette
ABCG: ABC transporter subfamily G
E-L: Eichorn and Lorenz growth scale
LOD: logarithm of odds
OSC: oxidosqualene cyclase
QTL: quantitative trait locus
VLCFA: very long chain fatty acid
VvTTPS: *Vitis vinifera* triterpene synthase
WSD1: wax synthase/diacylglycerol acyltransferase
Y1: year 1
Y2: year 2.
