# Supplementary material for: Metabolic and genetic analysis links TRITERPENE SYNTHASE 12 to oleanolic acid biosynthesis in grape berry wax
Source: J Exp Bot. 2025 Mar 18;76(11):3186–205. doi: 10.1093/jxb/eraf119 (PMC12321741; doi:10.1093/jxb/eraf119)
Supplement: eraf119_suppl_Supplementary_Tables_S1-S7_Figures_S1-S6 [file eraf119_suppl_supplementary_tables_s1-s7_figures_s1-s6.pdf]

Supplementary Data

Table S1: Summary of the samples analysed from the 'Deckrot' x G1-7720 family, indicating the relationship (parent or offspring), wax status, sample time and analysis.

Sample	Relationship	Wax status	Sampled at	Analysis
**DR_2**	parent	glaucous	Green, Véraison, Ripe	Wax characterisation
**DR_3**	parent	glaucous	Ripe	Wax characterisation, QTL
**DR_4**	parent	glaucous	Green, Véraison, Ripe	Wax characterisation, QTL
**G1_2**	parent	glossy	Green, Véraison, Ripe	Wax characterisation, QTL
**G1_3**	parent	glossy	Green, Véraison, Ripe	Wax characterisation
**G1_5**	parent	glossy	Ripe	Wax characterisation, QTL
**DG002**	offspring	glossy	Ripe	Wax characterisation, QTL
**DG007**	offspring	glossy	Ripe	Botrytis assay, Wax characterisation, QTL
**DG010**	offspring	intermediate	Ripe	Wax characterisation, QTL
**DG013**	offspring	glossy	Ripe	Wax characterisation, QTL
**DG016**	offspring	glaucous	Ripe	Wax characterisation, QTL
**DG021**	offspring	glossy	Ripe	Wax characterisation, QTL
**DG022**	offspring	glaucous	Ripe	Wax characterisation, QTL
**DG023**	offspring	intermediate	Ripe	Wax characterisation, QTL
**DG024**	offspring	intermediate	Ripe	Wax characterisation, QTL
**DG029**	offspring	glossy	Green, Véraison, Ripe	Wax characterisation
**DG032**	offspring	glossy	Ripe	Wax characterisation, QTL
**DG033**	offspring	glaucous	Green, Véraison, Ripe	Wax characterisation
**DG035**	offspring	glossy	Ripe	Wax characterisation, QTL
**DG037**	offspring	intermediate	Ripe	Wax characterisation, QTL
**DG040**	offspring	glossy	Ripe	Wax characterisation, QTL
**DG042**	offspring	glaucous	Ripe	Wax characterisation, QTL
**DG046**	offspring	glaucous	Ripe	Wax characterisation, QTL
**DG050**	offspring	glaucous	Ripe	Wax characterisation, QTL
**DG052**	offspring	intermediate	Ripe	Wax characterisation, QTL
**DG055**	offspring	glossy	Ripe	Botrytis assay, Wax characterisation, QTL
**DG057**	offspring	glossy	Ripe	Wax characterisation, QTL
**DG061**	offspring	glossy	Ripe	Wax characterisation, QTL
**DG062**	offspring	glaucous	Ripe	Wax characterisation, QTL
**DG063**	offspring	glaucous	Ripe	Wax characterisation, QTL
**DG064**	offspring	glossy	Ripe	Wax characterisation, QTL
**DG066**	offspring	glossy	Ripe	Wax characterisation, QTL
**DG067**	offspring	intermediate	Ripe	Wax characterisation, QTL
**DG071**	offspring	intermediate	Ripe	Wax characterisation, QTL
**DG079**	offspring	intermediate	Ripe	Wax characterisation, QTL
**DG081**	offspring	intermediate	Ripe	Wax characterisation, QTL
**DG085**	offspring	glaucous	Ripe	Wax characterisation, QTL
**DG086**	offspring	glossy	Ripe	Wax characterisation, QTL
**DG089**	offspring	glaucous	Ripe	Wax characterisation, QTL
**DG091**	offspring	glaucous	Ripe	Wax characterisation, QTL
**DG096**	offspring	glossy	Ripe	Wax characterisation, QTL
**DG097**	offspring	glossy	Ripe	Wax characterisation, QTL
**DG098**	offspring	glaucous	Ripe	Wax characterisation, QTL
**DG100**	offspring	glaucous	Ripe	Wax characterisation, QTL
**DG101**	offspring	glossy	Ripe	Wax characterisation, QTL
**DG102**	offspring	glossy	Ripe	Wax characterisation, QTL
**DG105**	offspring	glossy	Ripe	Wax characterisation, QTL
**DG106**	offspring	glossy	Ripe	Wax characterisation, QTL
**DG107**	offspring	glaucous	Ripe	Wax characterisation, QTL
**DG113**	offspring	glaucous	Ripe	Wax characterisation, QTL
**DG114**	offspring	glaucous	Ripe	Wax characterisation, QTL
**DG115**	offspring	intermediate	Ripe	Wax characterisation, QTL
**DG116**	offspring	glossy	Green, Véraison, Ripe	Wax characterisation, QTL
**DG118**	offspring	glaucous	Ripe	Wax characterisation, QTL
**DG122**	offspring	intermediate	Ripe	Wax characterisation, QTL
**DG124**	offspring	intermediate	Ripe	Wax characterisation, QTL

**DG125**	offspring	intermediate	Ripe	Wax characterisation, QTL
**DG126**	offspring	intermediate	Ripe	Wax characterisation, QTL
**DG127**	offspring	glossy	Ripe	Wax characterisation, QTL
**DG129**	offspring	glossy	Ripe	Wax characterisation, QTL
**DG130**	offspring	intermediate	Ripe	Wax characterisation, QTL
**DG133**	offspring	glaucous	Ripe	Botrytis assay, Wax characterisation, QTL
**DG135**	offspring	glossy	Green, Véraison, Ripe	Wax characterisation, QTL
**DG139**	offspring	intermediate	Ripe	Wax characterisation, QTL
**DG141**	offspring	glaucous	Ripe	Wax characterisation, QTL
**DG142**	offspring	glaucous	Ripe	Wax characterisation, QTL
**DG143**	offspring	intermediate	Ripe	Wax characterisation, QTL
**DG144**	offspring	glaucous	Ripe	Wax characterisation, QTL
**DG145**	offspring	intermediate	Ripe	Wax characterisation, QTL
**DG147**	offspring	intermediate	Ripe	Wax characterisation, QTL
**DG151**	offspring	intermediate	Ripe	Wax characterisation, QTL
**DG154**	offspring	glaucous	Ripe	Wax characterisation, QTL
**DG155**	offspring	glossy	Ripe	Wax characterisation, QTL
**DG157**	offspring	intermediate	Ripe	Wax characterisation, QTL
**DG162**	offspring	glossy	Ripe	Wax characterisation, QTL
**DG166**	offspring	intermediate	Ripe	Wax characterisation, QTL
**DG170**	offspring	intermediate	Ripe	Wax characterisation, QTL
**DG171**	offspring	glaucous	Green, Véraison, Ripe	Wax characterisation
**DG172**	offspring	glaucous	Green, Véraison, Ripe	Wax characterisation, QTL
**DG173**	offspring	glaucous	Ripe	Wax characterisation, QTL
**DG175**	offspring	glaucous	Ripe	Wax characterisation, QTL
**DG176**	offspring	intermediate	Ripe	Wax characterisation, QTL
**DG177**	offspring	glossy	Ripe	Wax characterisation, QTL
**DG179**	offspring	intermediate	Ripe	Wax characterisation, QTL
**DG182**	offspring	glossy	Ripe	Wax characterisation, QTL
**DG183**	offspring	intermediate	Ripe	Wax characterisation, QTL
**DG186**	offspring	glossy	Ripe	Wax characterisation, QTL
**DG187**	offspring	intermediate	Ripe	Wax characterisation, QTL
**DG188**	offspring	glaucous	Ripe	Botrytis assay, Wax characterisation, QTL
**DG191**	offspring	intermediate	Ripe	Wax characterisation, QTL
**DG197**	offspring	intermediate	Ripe	Wax characterisation, QTL
**DG205**	offspring	glaucous	Ripe	Wax characterisation, QTL
**DG208**	offspring	intermediate	Ripe	Wax characterisation, QTL
**DG212**	offspring	intermediate	Ripe	Wax characterisation, QTL
**DG215**	offspring	glaucous	Ripe	Wax characterisation, QTL
**DG222**	offspring	intermediate	Ripe	Wax characterisation, QTL
**DG223**	offspring	glossy	Ripe	Botrytis assay, Wax characterisation, QTL
**DG224**	offspring	glaucous	Ripe	Wax characterisation, QTL
**DG225**	offspring	glaucous	Ripe	Botrytis assay, Wax characterisation, QTL

Table S2: Compounds occurring in the cuticular wax of grapevine berries as determined by GC-MS.

Peak	Compound	Min RT	Max RT	Fragments (m/z)	Chain length	Compound type
1	C16 fatty acid	14.50	14.65	73, 117, 313, 328	C16	Fatty acid
2	C18 linoleic acid (C18:2)	17.03	17.34	73, 81, 129, 337	C18	Fatty acid
3	C18 oleic acid (C18:1)	17.03	17.36	73, 117, 339	C18	Fatty acid
4	C18 stearic acid (C18:0)	17.63	17.84	73, 117, 341, 356	C18	Fatty acid
5	C24 alkane (IS)	20.11	20.29	57, 71, 85, 338	C24	Internal standard
6	unknown1	20.13	20.36	225, 369, 440		Unknown
7	C20 fatty acid	20.69	20.81	73, 117, 369, 384	C20	Fatty acid
8	unknown2	21.14	21.26	314, 385		Unknown
9	C25 alkane	21.58	21.76	57, 71, 85, 352	C25	Alkane
10	C22 alcohol	22.26	22.56	75, 103, 383	C22	Alcohol
11	C24 aldehyde	24.06	24.20	82, 96, 110, 334	C24	Aldehyde
12	C22 fatty acid	24.00	24.27	117, 129, 1325, 145, 397, 412	C22	Fatty acid
13	C27 alkane	25.10	25.33	57, 71, 85, 380	C27	Alkane
14	C25 aldehyde	25.67	25.82	82, 96, 110, 348	C25	Aldehyde
15	C24 alcohol	25.78	25.88	75, 103, 411	C24	Alcohol
16	C24 fatty acid	26.66	26.86	73, 75, 117, 129, 132, 145, 381, 425	C24	Fatty acid
17	C26 aldehyde	26.69	26.85	82, 96, 110, 362	C26	Aldehyde
18	C25 alcohol	26.67	26.80	75, 103, 207, 281, 425, 482	C25	Alcohol
19	C29 alkane	27.16	27.23	57, 71, 85, 408, 474	C29	Alkane
20	C25 fatty acid	27.19	27.67	117, 129, 132, 145, 439, 454	C25	Fatty acid
21	C26 alcohol	27.38	27.67	75, 103, 440, 585	C26	Alcohol
22	C26 fatty acid	27.84	28.08	73, 117, 132, 145, 453, 468	C26	Fatty acid
23	C28 aldehyde	28.07	28.14	82, 96, 390, 408	C28	Aldehyde
24	C31 alkane	28.41	28.67	57, 71, 85, 436	C31	Alkane
25	C28 alcohol	28.64	28.71	73, 75, 103, 468	C28	Alcohol
26	Cholestanol	28.75	28.84	107, 2155, 445, 460		Internal standard
27	C28 fatty acid	29.24	29.32	73, 117, 481, 496	C28	Fatty acid
28	C30 aldehyde	29.35	29.44	82, 96, 110, 418	C30	Aldehyde
29	C30 alcohol	29.95	30.04	73, 75, 103, 496	C30	Alcohol
30	unknown fatty acid1	29.97	30.09	73, 75, 207, 357, 486		Fatty acid
31	unknown3	30.03	30.18	189, 204, 281		Unknown
32	unknown4	29.91	30.22	73, 75, 189, 204		Unknown
33	β-amyrin	30.21	30.32	73, 75, 203, 218		Triterpene
34	unknown5	30.22	30.58	75, 107, 255, 486		Unknown
35	α-amyrin	30.48	30.78	73, 281, 393		Triterpene
36	C30 fatty acid	30.72	30.93	73, 117, 509, 524	C30	Fatty acid
37	C32 aldehyde	30.91	31.15	82, 96, 110, 446	C32	Aldehyde
38	unknown6	31.04	31.35	73, 379, 570		Unknown
39	unknown7	31.31	31.43	73, 203, 216, 496		Unknown
40	unknown8	31.44	31.57	202		Unknown
41	C32 alcohol	31.61	31.89	75, 103, 524	C32	Alcohol
42	Oleanolic acid	31.94	32.11	189, 203, 320, 182		Triterpene
43	OAderiv1	31.92	32.64	203, 320, 482		Triterpene
44	OAderiv2	32.25	32.49	73, 75, 203, 232		Triterpene
45	OAderiv3	32.25	32.59	203, 289, 320		Triterpene
46	C32 fatty acid	32.60	32.74	117, 129, 132, 145, 537	C32	Fatty acid
47	OAderiv4	32.72	32.97	189, 203, 320, 355		Triterpene

Table S3: Significant concentration differences of grape berry cuticular wax compounds between the different developmental stages. The columns indicate the average compound abundances at the various stages (in ng.mm^{-2}), as well as the Kruskal-Wallis *P*-value and pairwise Wilcoxon comparison *P*-values between the different stages. Significant *P*-values are indicated in bold and red.

	Averages (ng.mm^{-2})			*P*-values			
	**Green**	**Véraison**	**Ripe**	**Kruskal-Wallis**	**Green vs Véraison**	**Véraison vs Ripe**	**Green vs Ripe**
C18:2 fatty acid	0.70	0.34	0.21	**0.0002**	**0.0052**	**0.0068**	**0.0002**
C20 fatty acid	0.13	0.21	0.38	**0.0007**	**0.0232**	**0.0147**	**0.0003**
C22 fatty acid	0.24	0.24	0.42	**0.0028**	**0.9118**	**0.0011**	**0.0039**
C26 fatty acid	2.48	3.29	4.24	**0.0137**	**0.0892**	**0.1903**	**0.0039**
C28 fatty acid	0.76	1.58	2.38	**0.0000**	**0.00001**	**0.0068**	**0.00001**
unknown3	0.49	0.18	0.1	**0.0015**	**0.0052**	**0.6842**	**0.0002**
unknown4	0.01	0.02	0.03	**0.0269**	**0.0450**	**0.6310**	**0.0140**
C30 fatty acid	0.69	1.29	1.6	**0.0001**	**0.3332**	**0.1230**	**0.00002**
C32 aldehyde	0.28	0.19	0.16	**0.0238**	**0.0524**	**0.4813**	**0.0089**
unknown9	0.05	0.02	0.01	**0.0002**	**0.0005**	**0.3527**	**0.00001**
C32 fatty acid	0.16	0.04	0.02	**0.0002**	**0.0003**	**0.9705**	**0.00001**
Total fatty acids	10.93	11.74	13.95	**0.0164**	**0.3930**	**0.0288**	**0.0089**
Fatty acids:Unknown	1.8	1.99	2.76	**0.0269**	**0.2560**	**0.0630**	**0.0170**
Alkanes:Aldehydes	0.2	0.28	0.37	**0.0461**	**0.0750**	**0.4270**	**0.0260**
Alcohols:Aldehydes	3.42	3.98	4.23	**0.0074**	**0.0091**	**0.3254**	**0.0101**
C20	0.13	0.21	0.38	**0.0007**	**0.0188**	**0.0230**	**0.0010**
C28	4.89	5.45	6.31	**0.0494**	**0.2730**	**0.1230**	**0.0260**
C30	2.12	2.62	2.89	**0.0038**	**0.0190**	**0.1618**	**0.0032**
C32	0.77	0.49	0.40	**0.0047**	**0.0172**	**0.5705**	**0.0022**
Cuticle Weight (mg)	7.04	4.63	4.12	**0.0161**	**0.0255**	**0.5197**	**0.0089**

Table S4: Significant differences in grape berry cuticular wax compound concentrations between year 1 (Y1) and year 2 (Y2) in the 'Deckrot' parent. The columns indicate the average compound abundances of Y1 and Y2 (in ng.mm^{-2}), the P-value and contribution of the compound to the wax difference between the two years.

	Average Y1	Average Y2	*P*-value	Contribution
**Total wax**	1342.95	845.89	0.0204	100%
**Oleanolic acid**	853.75	596.76	0.0483	52%
**Total fatty acids**	186.18	83.07	0.0200	21%
**Total alcohols**	140.58	48.71	0.0190	18%
**C26**	132.18	45.66	0.0145	17%
**Total unknowns**	96.12	19.14	0.0131	15%
**C28**	72.82	27.24	0.0095	9%
**C26 fatty acid**	60.91	23.01	0.0207	8%
**C24**	52.51	17.05	0.0096	7%
**Total aldehydes**	43.46	14.41	0.0111	6%
**C28 alcohol**	41.30	15.70	0.0121	5%
**C24 alcohol**	27.77	9.56	0.0061	4%
**C24 fatty acid**	21.39	6.68	0.0364	3%
**C28 fatty acid**	21.64	8.34	0.0293	3%
**C26 aldehyde**	20.60	7.44	0.0126	3%
**C30 fatty acid**	14.84	6.05	0.0264	2%
**C30 alcohol**	11.78	4.65	0.0087	1%
**C28 aldehyde**	9.88	3.20	0.0158	1%
**C25**	8.90	2.68	0.0083	1%
**C22**	7.63	2.58	0.0290	1%
**Total alkanes**	8.26	3.50	0.0273	1%
**C22 fatty acid**	5.56	1.63	0.0265	1%
**C30 aldehyde**	5.39	1.62	0.0114	1%
**C32**	7.32	3.61	0.0269	1%
**β-amyrin**	14.23	11.18	0.0295	1%
**C24 aldehyde**	3.35	0.81	0.0056	1%
**C25 alcohol**	3.47	1.06	0.0065	0%
**C32 aldehyde**	3.41	1.09	0.0121	0%
**C32 alcohol**	3.53	1.57	0.0090	0%
**C25 alkane**	2.90	0.99	0.0151	0%
**C25 fatty acid**	1.70	0.38	0.0155	0%
**C31 alkane**	1.64	0.89	0.0414	0%
**C31**	1.64	0.89	0.0414	0%
**C25 aldehyde**	0.83	0.25	0.0067	0%
**unknown4**	0.10	0.13	0.0098	0%
**unknown13**	0.04	0.16	0.0042	0%
**unknown9**	0.19	0.62	0.0012	0%

Table S5: Annotated genes in significant QTL regions. Putative candidate genes are indicated in green and bold.

Table S5 available at JXB online.

Table S6: Candidate grapevine triterpene synthase genes from the literature (*VvTTPS1-10*) as well as three additional putative terpene synthases (*VvTTPS11-13*) and three cycloartenol synthases (*VvCAS1_1-3*) as identified from homology searches in this study.

Name	Gene ID	Position	Reference	QTL in this study
VvTTPS1	Vitvi09g02006	chr09:22305271...22316730	Pensec *et al.*, 2016	QTL9.1
VvTTPS2	Vitvi09g01429	chr09:22154680...22159973	Pensec *et al.*, 2016	QTL9.1
VvTTPS3a	Vitvi09g01446	chr09:22368807...22376060	Pensec *et al.*, 2016	QTL9.1
VvTTPS3b	Vitvi09g02010	chr09:22366563...22368806	Pensec *et al.*, 2016	QTL9.1
VvTTPS5	Vitvi09g02008	chr09:22339721...22345662	Pensec *et al.*, 2016	QTL9.1
VvTTPS6	Vitvi09g02012	chr09:22441827...22446947	Pensec *et al.*, 2016	QTL9.1
VvTTPS7	Vitvi10g01875	chr10:9702059...9708331	Pensec *et al.*, 2016	-
VvTTPS8	Vitvi10g01862	chr10:9498414...9505286	Pensec *et al.*, 2016	-
VvTTPS9	Vitvi10g01863	chr10:9521908...9527534	Pensec *et al.*, 2016	-
VvTTPS10	Vitvi11g00878	chr11:11479515...11493491	Pensec *et al.*, 2016	-
VvTTPS11	Vitvi09g01426	chr09:22112718...22118770	This study	QTL9.1
VvTTPS12	Vitvi09g01427	chr09:22125467...22133940	This study	QTL9.1
VvTTPS13	Vitvi10g01876	chr10:9739343...9744311	This study	-
VvCAS1_1	Vitvi09g01453	chr9:22497270..22512320	This study	QTL9.1
VvCAS1_2	Vitvi09g01413	chr9:21957153..21975876	This study	QTL9.1
VvCAS1_3	Vitvi09g01414	chr9:21988345..22006054	This study	QTL9.1

Table S7: Functionally characterized plant terpene synthase genes included in the phylogenetic analysis of the grapevine terpene synthases.

Species	**Accession ID**	**Gene**	**Product**
Arabidopsis thaliana	At2g07050	*CAS1*	Cycloartenol
Arabidopsis thaliana	At3g45130	*LSS1*	Lanosterol
Arabidopsis thaliana	At1g78950	*LUP4*	β-amyrin
Arabidopsis thaliana	At1g66960	*LUP5*	Mixed products
Arabidopsis thaliana	At1g78960	*LUP2*	Mixed products
Arabidopsis thaliana	At1g78970	*LUP1*	Mixed products
Arabidopsis thaliana	At4g15370	*BARS1*	Mixed products
Arabidopsis thaliana	At4g15340	*PEN1*	Mixed products
Arabidopsis thaliana	At5g36150	*PEN3*	Mixed products
Arabidopsis thaliana	At1g78500	*PEN6*	Mixed products
Arabidopsis thaliana	At1g78955	*CAMS1*	Mixed products
Arabidopsis thaliana	At5g48010	*THAS1*	Thalianol
Arabidopsis thaliana	At5g42600	*MRN1*	Marneral
Panax ginseng	AB009031	*PNZ1*	Lanosterol
Panax ginseng	AB009029	*PNX*	Cycloartenol
Panax ginseng	AB009030.1	*PNY1*	β-amyrin
Panax ginseng	AB014057.1	*PNY2*	β-amyrin
Lotus japonicus	AB244671	*LAS*	Lanosterol
Lotus japonicus	AB181246	*OSC5*	Cycloartenol
Lotus japonicus	AB181244	*AMY1*	β-amyrin
Lotus japonicus	AB181245	*OSC3*	Lupeol
Lotus japonicus	AF478455.1	*AMY2*	Mixed products
Pisum sativum	D89619	*CASPEA*	Cycloartenol
Pisum sativum	AB034803.2	*PSM*	Mixed products
Pisum sativum	AB034802.1	*PSY*	β-amyrin
Solanum lycopersicum	HQ266579	*SlTTS1*	β-amyrin
Solanum lycopersicum	HQ266580	*SlTTS2*	Mixed products
Olea europaea	AB025343	*OEW*	Lupeol
Olea europaea	AB291240	*OEA*	α-amyrin
Betula platyphylla	AB055509	*BPX1*	Cycloartenol
Betula platyphylla	AB055510	*BPX2*	Cycloartenol
Betula platyphylla	AB055512	*BPY*	β-amyrin
Betula platyphylla	AB055511	*BPW*	Lupeol
Glycyrrhiza glabra	AB025968	*CAS1*	Cycloartenol
Glycyrrhiza glabra	AB037203	*bAS1*	β-amyrin
Glycyrrhiza glabra	AB116228	*LUS1*	Lupeol

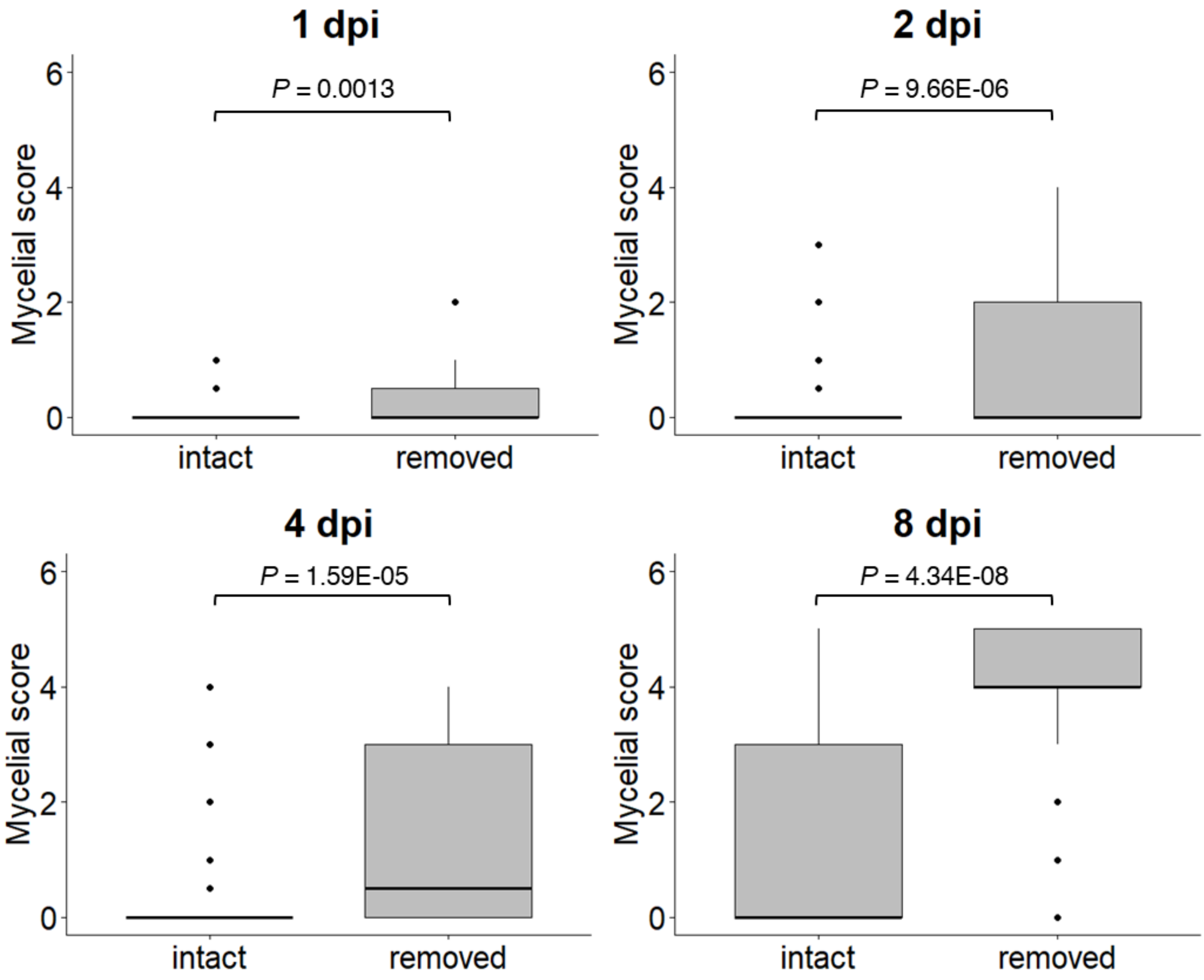

Figure S1: Comparison of mycelial growth scores (indication of infection) at 1 dpi, 2 dpi, 4 dpi and 8 dpi between berries with wax intact or wax removed. Mycelial growth was scored on a scale of 0 (no growth) to 5 (berry densely covered with mycelia). dpi, days post infection.

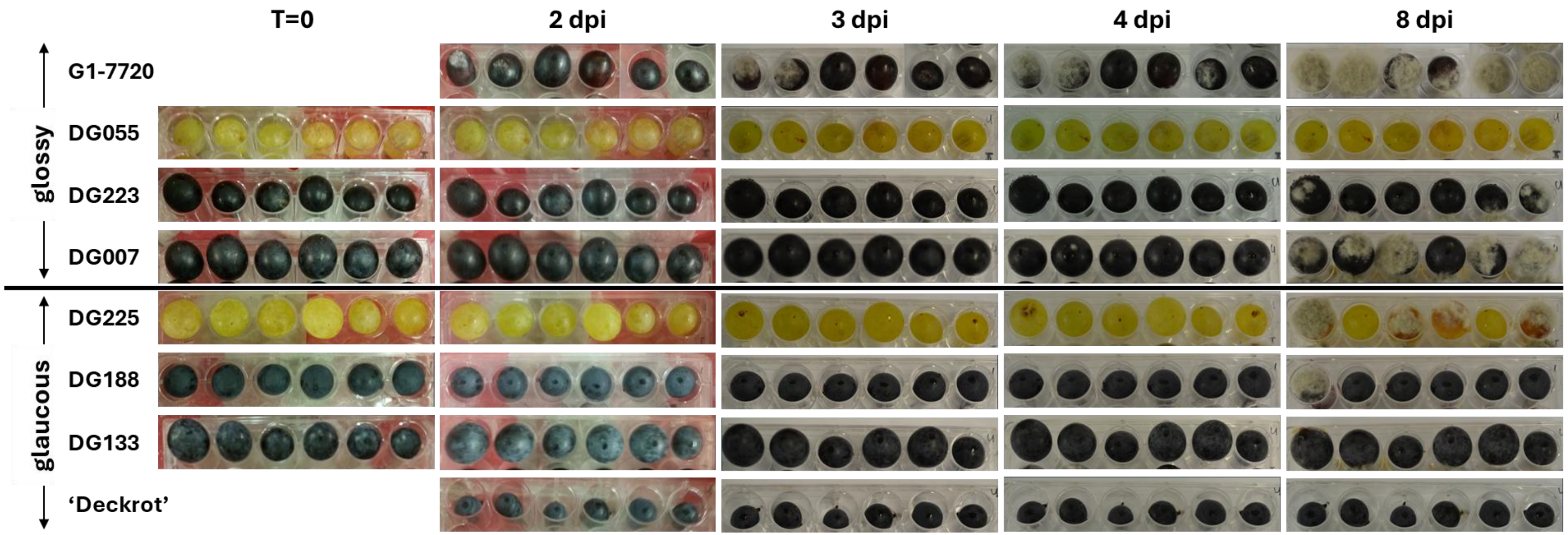

Figure S2: *Botrytis cinerea* infections in 'Deckrot', a glaucous grape variety, and G1-7720, a glossy grape accession, as well as three glossy offspring (DG055, DG223, DG007) and three glaucous offspring (DG225, DG188, DG133) over 8 days. This figure is an extension of Figure 2 and shows the infection of wax+ individuals over the time course of the experiment. dpi, days post infection.

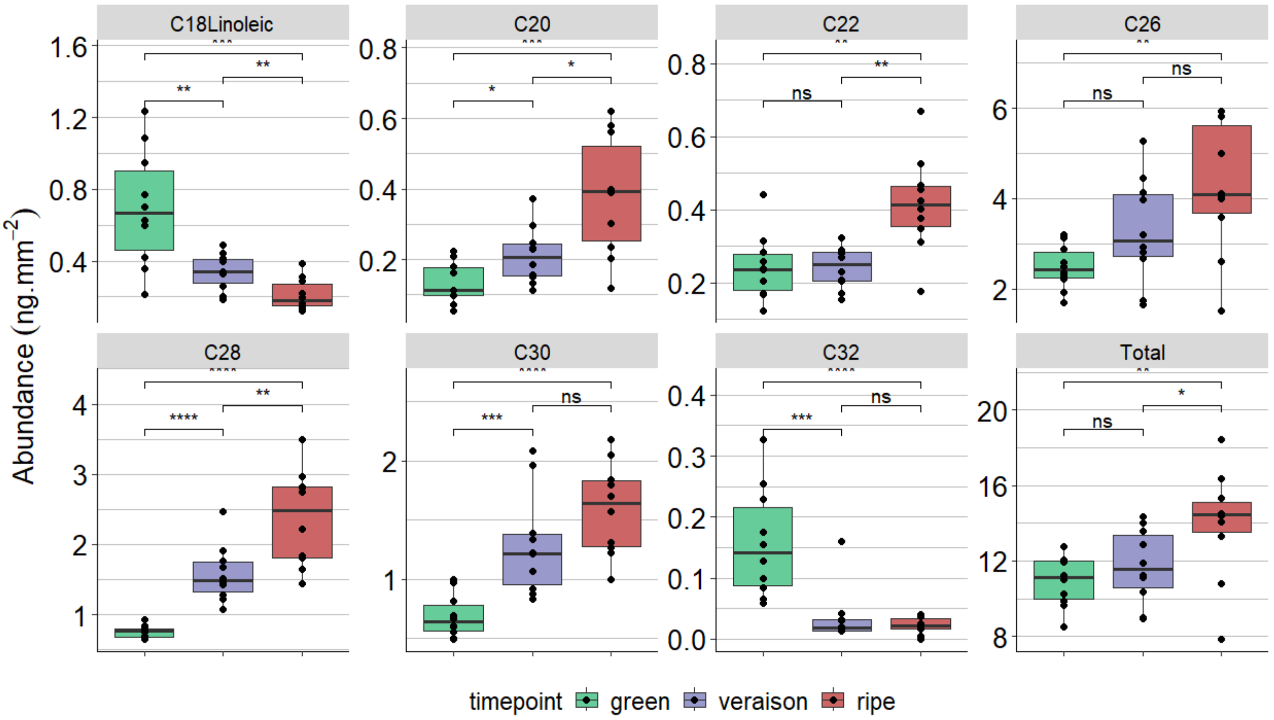

Figure S3: Comparison of the relative abundance of various fatty acids in grape berry cuticular waxes between the green (n=8), véraison (n=8) and ripe (n=8) stages. Relative abundance is indicated in ng.mm^{-2}. The group means of each stage were compared and significant differences between groups are indicated. *, $P < 0.05$; **, $P < 0.01$; ***, $P < 0.001$; ****, $P < 0.0001$; ns, not significant.

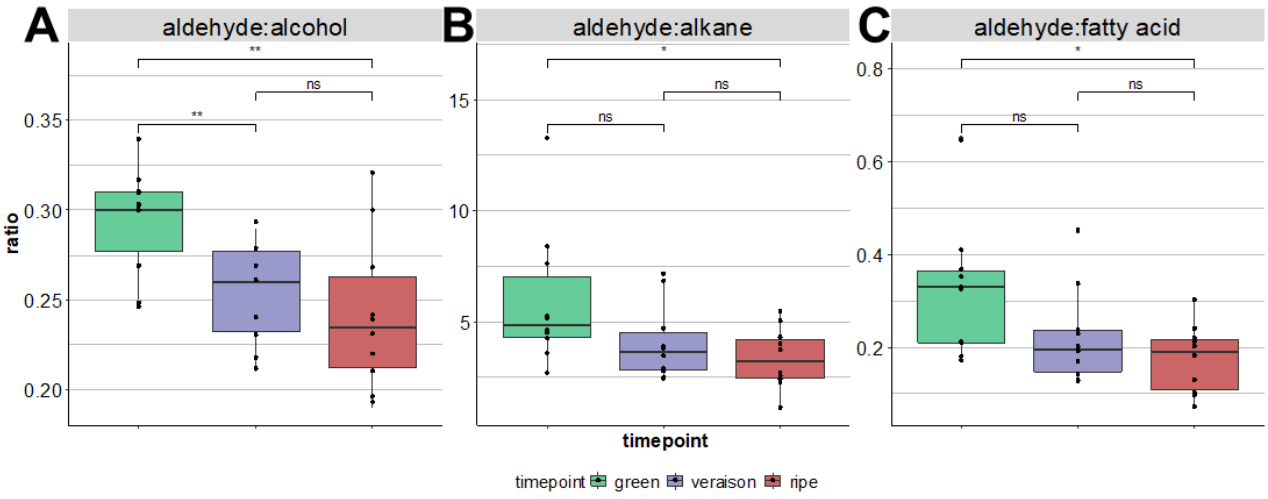

Figure S4: Comparison across the green, véraison and ripe stages of grape berries for the ratio of aldehydes versus A) alcohols, B) alkanes and C) fatty acids in cuticular wax. Relative abundance is indicated in ng.mm^{-2}. The group means of each stage were compared and significant differences between groups are indicated. *, P < 0.05; **, P < 0.01; ***, P < 0.001; ****, P < 0.0001; ns, not significant.

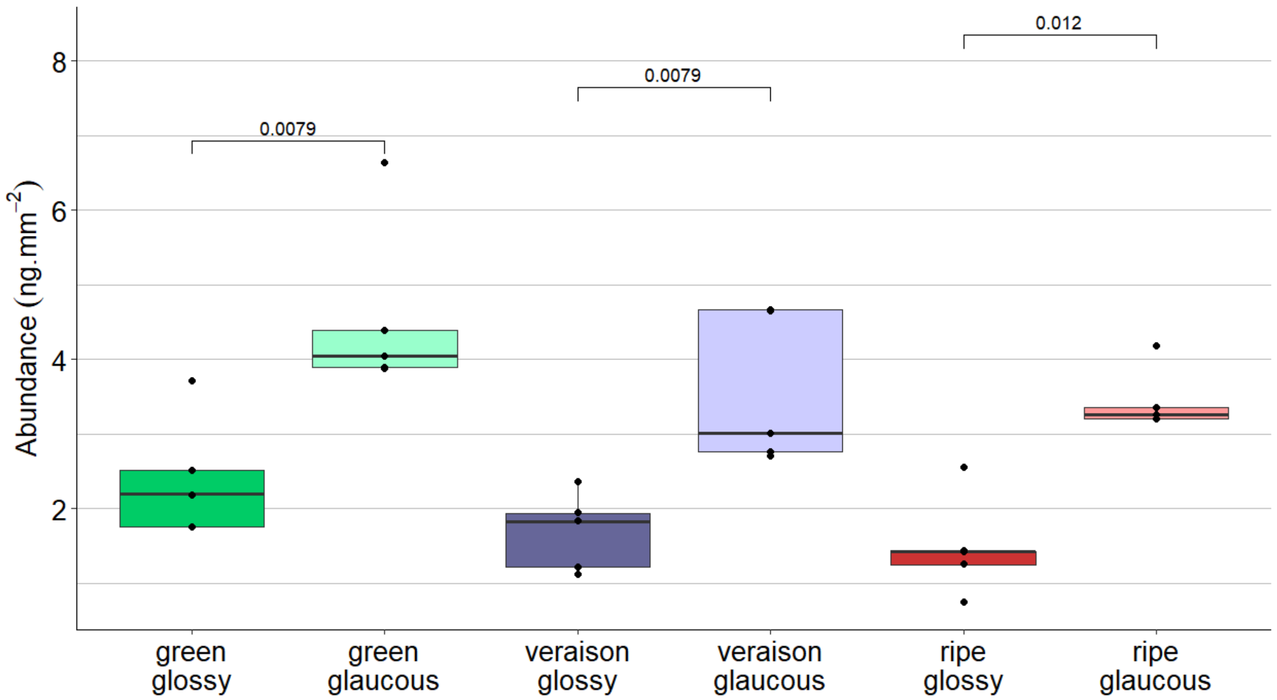

Figure S5: Comparison of the total aldehydes in the grape berry cuticular wax between the glossy (n=4) and glaucous (n=4) phenotypes for the green, véraison and ripe stages. Relative abundance is indicated in ng.mm^{-2}. Group means between glossy and glaucous phenotypes were compared and *P*-values are indicated.

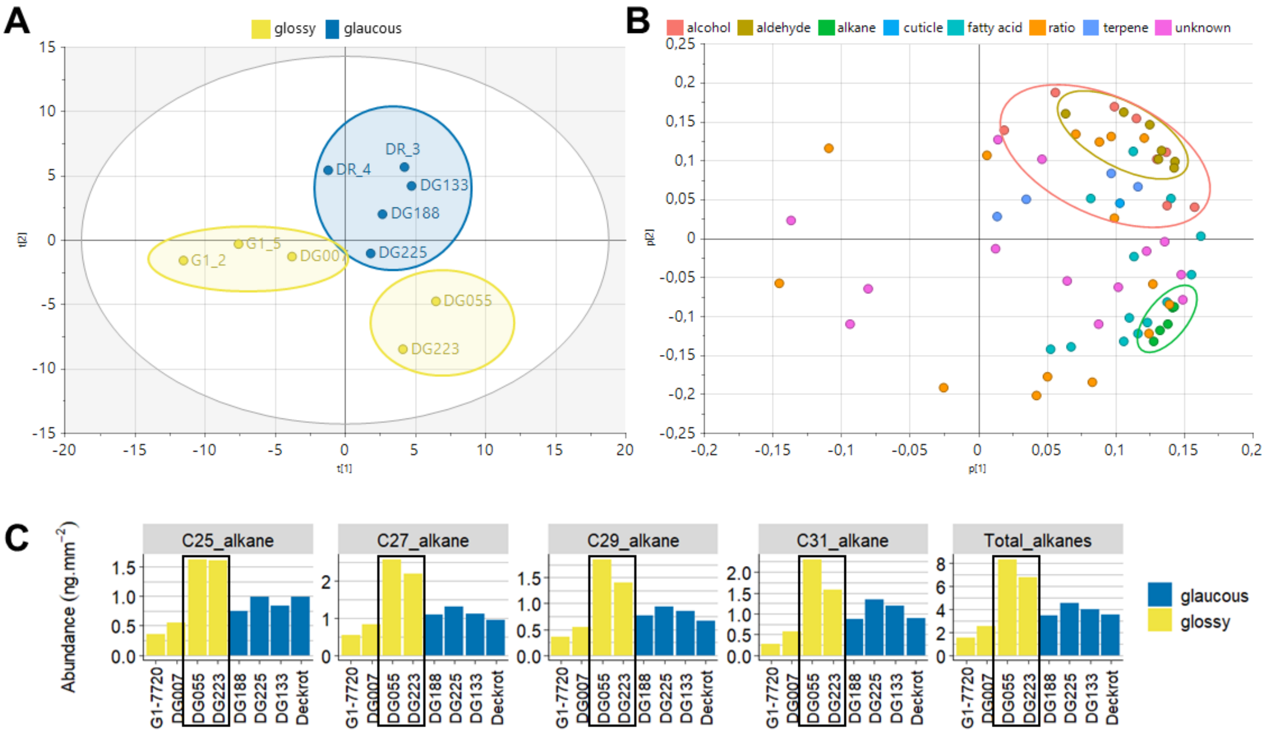

Figure S6: Compositional differences in cuticular wax of grape berries and *Botrytis cinerea* susceptibility, as measured in the parents ('Deckrot' and G1-7720) and glossy (n=3) and glaucous (n=3) seedlings. The **(A)** score and **(B)** loadings plot of the principal component analysis indicated an association of alkanes with the glossy resistant individuals DG055 and DG223. Circles indicate the clustering of the alcohol, aldehyde and alkane compounds. **(C)** All alkanes were increased in these two individuals.
